# Using conjoint analysis to develop a system to score research engagement actions by health decision makers

**DOI:** 10.1186/s12961-015-0013-z

**Published:** 2015-04-26

**Authors:** Steve R Makkar, Anna Williamson, Tari Turner, Sally Redman, Jordan Louviere

**Affiliations:** 1 The Sax Institute, Level 13, Building 10, 235 Jones Street, Ultimo, New South Wales 2007 Australia; 2 World Vision Australia, 1 Vision Drive, Burwood East, Victoria 3151 Australia; 3 School of Marketing, University of South Australia, Level 4, Yungondi Building, North Terrace, Adelaide, South Australia 5000 Australia

**Keywords:** Conjoint analysis, Evidence-based policy, Evidence-informed policy, Health policy, Knowledge translation, Measurement, Policymaker, Utilisation

## Abstract

**Background:**

Effective use of research to inform policymaking can be strengthened by policymakers undertaking various research engagement actions (e.g., accessing, appraising, and applying research). Consequently, we developed a thorough measurement and scoring tool to assess whether and how policymakers undertook research engagement actions in the development of a policy document. This scoring tool breaks down each research engagement action into its key ‘subactions’ like a checklist. The primary aim was to develop the scoring tool further so that it assigned appropriate scores to each subaction based on its effectiveness for achieving evidence-informed policymaking. To establish the relative effectiveness of these subactions, we conducted a conjoint analysis, which was used to elicit the opinions and preferences of knowledge translation experts.

**Method:**

Fifty-four knowledge translation experts were recruited to undertake six choice surveys. Respondents were exposed to combinations of research engagement subactions called ‘profiles’, and rated on a 1–9 scale whether each profile represented a limited (1–3), moderate (4–6), or extensive (7–9) example of each research engagement action. Generalised estimating equations were used to analyse respondents’ choice data, where a utility coefficient was calculated for each subaction. A large utility coefficient indicates that a subaction was influential in guiding experts’ ratings of extensive engagement with research.

**Results:**

The calculated utilities were used as the points assigned to the subactions in the scoring system. The following subactions yielded the largest utilities and were regarded as the most important components of engaging with research: searching academic literature databases, obtaining systematic reviews and peer-reviewed research, appraising relevance by verifying its applicability to the policy context, appraising quality by evaluating the validity of the method and conclusions, engaging in thorough collaborations with researchers, and undertaking formal research projects to inform the policy in question.

**Conclusions:**

We have generated an empirically-derived and context-sensitive method of measuring and scoring the extent to which policymakers engaged with research to inform policy development. The scoring system can be used by organisations to quantify staff research engagement actions and thus provide them with insights into what types of training, systems, and tools might improve their staff’s research use capacity.

**Electronic supplementary material:**

The online version of this article (doi:10.1186/s12961-015-0013-z) contains supplementary material, which is available to authorized users.

## Background

Health policymaking is a complex process, influenced by a vast number of factors within the decision-making context such as political pressures, stakeholder interests, feasibility aspects, and numerous sources of information (e.g., past policy documents, internal program evaluations) [1,2]. In recent times, however, there have been calls for governments and health systems worldwide to ensure that health policies are also informed by research evidence in order to improve the likelihood that state and national objectives for improved health and efficient health spending are achieved [2-5].

Although numerous studies have demonstrated a link between the implementation of evidence-informed policies and improvements in health [6-10], evidence suggests that, globally, there is still a considerable gap between evidence of effective strategies and the health policies that are developed and implemented [3,6,11-14]. Indeed, numerous studies have shown that a considerable proportion of health policymakers do not routinely use research to inform policy development [15-20].

Evidence suggests that this research to policy gap is, in part, due to barriers affecting policymakers’ capacity to access research (i.e., search for and retrieve research to inform policy), appraise research (i.e., evaluate its scientific quality and relevance to the policy issue and context), generate new research or analyses (e.g., externally commission research or conduct research internally), and/or interact with researchers (e.g., communicate, consult, and/or collaborate with relevant researchers) [16,21-30]. The Supporting Policy in Health with Research: an Intervention Trial (SPIRIT) Action Framework [19] – a conceptual model developed to inform a multifaceted program to increase the capacity of policy agencies to use research – collectively refers to these actions of accessing, appraising, and/or generating relevant and high-quality research evidence as research engagement actions. These research engagement actions are distinct from what is sometimes termed research engagement, which describes policymakers’ awareness and perceptions of the value of research, and generating interest and dialogue between the public and research community [31].

### The importance of measuring research engagement actions and existing measures

As government leaders worldwide have pledged to increase the extent to which evidence underpins their policies, pressure has increased on the policy workforce to strengthen their capacity to transfer research evidence into policy [31,32]. It is useful, therefore, to measure policymakers’ research engagement actions because the ability of policymakers to use research to inform policy is directly dependent on performing at least some of these actions. At the most basic level, if research is neither searched for nor accessed then it cannot be used to inform policymaking. Further, if research is not appraised, then even if research evidence is used in policy development, the quality of that research may not be optimal. Measures of research engagement actions can be used to evaluate policymakers’ and organisations’ current capacities to engage with research, evaluate whether their methods of engaging with research are optimal (e.g., whether they are searching appropriate evidence sources and databases to find relevant research to inform policy), and to highlight key areas where skills building may be required. Such measures can also be used to assess the impact of organisational initiatives or programs to improve these skills or capacities [32].

Unfortunately, no comprehensive measures of research engagement actions are currently available [33]. Current measures at most assess one or two research engagement actions (e.g., interactions with researchers [15,34], accessing research [35], or appraising research [15]), are primarily self-reported, and measure research engagement actions very generally and not in relation to specific policies or programs. Zardo and Collie [35], however, developed a content analysis method where discrete policy documents are examined and coded for type of evidence cited (e.g., academic evidence, internal policies, medical evidence). Unfortunately, their measurement approach has limitations because it does not take into account research that was not cited, but which nonetheless contributed to the development of the policy document, either directly or indirectly [36].

### SAGE: A new measure of research engagement actions

To overcome the above-mentioned limitations, we developed the Staff Assessment of enGagement with Evidence from research (SAGE) – a comprehensive, theory-based, and multi-modal measure of policymakers’ research engagement actions and research use in the development of a policy document. SAGE was developed by the Centre for Informing Policy in Health with Evidence from Research (CIPHER). CIPHER was established with the aims of developing and testing new strategies to increase the use of research evidence in all aspects of policymaking (i.e., agenda-setting, development, implementation, and evaluation [19]), improving policymakers’ access to information summaries, building researchers’ skills in working with policy agencies, and developing new ways of measuring the use of research in policy.

SAGE is informed by the SPIRIT Action Framework [19], which does not assume that policymaking is a linear, predictable process, but simply provides a simplified schematic to summarise the process through which research informs policymaking. Specifically, the framework describes how once research is needed to inform policy, policymakers initiate a number of research engagement actions, such as i) searching for and ii) obtaining research; iii) appraising its relevance to the policy issue and iv) its quality in terms of methodological rigour and validity; v) generating new research and/or data analyses; and vi) interacting with researchers. Once relevant research has been obtained and/or generated as a consequence of these actions, it can then be used in four different ways to inform policymaking (i.e., research use actions). Specifically, research may be used to directly inform decisions relating to the identified policy issue(s) (instrumental use; [15,37,38]), clarify understanding about the policy issue without directly influencing the decision (conceptual use [39-41]), justify and/or persuade others to support a predetermined decision (tactical use [42,43]), or be used to meet legislative, funding, or organisational requirements (imposed use). Ultimately, the framework predicts that research use will lead to more evidence-informed policies, and ultimately better health services and outcomes.

Informed by this framework, SAGE broadly assesses i) the extent to which policymakers undertook research engagement actions, and ii) the extent to which research was used to inform the development of a policy document. The majority of SAGE data are collected via a structured, qualitative interview regarding a specific policy document and the process undertaken to develop it. The interview is conducted with a person who was heavily involved in the document’s development. In the SAGE interview, policymakers are asked to describe the research engagement actions they undertook in developing the policy document (i.e., how research was searched for, obtained, appraised, generated) and if and how research was ultimately used to inform the document (see Additional file 1 for the SAGE interview). The interview takes approximately 40 min to complete, and is administered by a trained interviewer with experience in interviewing and qualitative analysis.

The SAGE interview thus captures contextual details, as well as the diverse range of processes, research engagement actions, and uncited research that contributed to the development of the document. To complement this information, reference lists associated with the document (if available) are scanned to identify the types of research that directly informed the document. It should be noted that an absence of cited research does not imply that research was not used.

**Figure 1:**
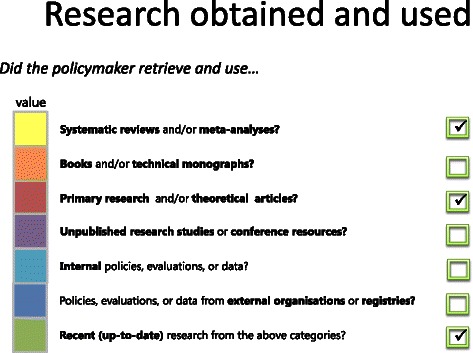
Example checklist for scoring the types of research obtained and used.

SAGE includes a scoring tool which allows the conversion of the interview data into a quantitative format. The scoring tool consists of a comprehensive checklist that breaks down each research engagement action into its main subactions. These subactions are the essential features or actions of each research engagement action, identified from literature on evidence-informed policymaking as well as data obtained from SAGE interviews undertaken with policymakers (see Methods for details). For example, the subactions of searching for research include searching academic literature databases or libraries and searching sources of grey literature (see Table 1 for definitions of key terms and examples). The scoring tool defines each subaction in concrete detail and provides examples to aid scoring. If, on the basis of the interview transcript and policy document, the scorer judges that a subaction was performed in the development of the policy document it is ticked off on the scoring tool (See Figure 1 for an example). The scoring tool thus provides a systematic means of documenting and describing the range of subactions underpinning each research engagement action undertaken during the development of the policy development.

**Table 1:** **Definitions of key terms**

Term	Definition	Example
Research engagement actions	Actions undertaken by policymakers to acquire, appraise, and generate relevant and high-quality research evidence or information to inform policymaking.	Examples of research engagement actions include 1) searching for and 2) obtaining research, 3) appraising the relevance and 4) quality of research, 5) generating new research or data analyses, and 6) interacting with researchers to acquire research-related information. The SAGE scoring tool addresses these six research engagement actions.
Subactions	Subactions^†^ are the essential features or main actions of each research engagement action. They often refer to broad, concrete example actions of undertaking each research engagement action. Each research engagement has a number of subactions that were identified through examination of literature on evidence-informed policymaking and interviews with policymakers.	Examples of subactions of searching for research include a) searching academic literature databases or libraries; b) searching sources of grey literature; c) identifying research by chance, using on-hand research, or research being provided by colleagues; d) seeking out experts to search for relevant research; e) searching for research in search engines or social media sharing sites; and f) examining reference lists, citation indices or databases of references.
	^†^In order to enhance clarity and comprehension throughout the paper, we used the term subaction instead of attribute, which is most commonly used in choice studies and conjoint analysis.	
Level	Levels in conjoint analysis refer to all the possible values of a subaction and are often described in concrete terms. To undertake a conjoint analysis, each subaction must be divided into concrete, perceptible levels. In the present study, the majority of subactions were divided into two levels: i) yes, the subaction was performed by the policymaker, or ii) no, it was not performed by the policymaker. Different levels of subactions are combined in various combinations to form ‘profiles’.	As above, one of the subactions of searching for research was ‘searching academic literature databases’. This subaction has two levels: i) yes, the policymaker searched for research in academic literature databases, or ii) no, the policymaker did not search academic literature databases.
Profile	A research engagement action profile is made up of a combination of subaction levels. Specifically, a profile consists of one level of each subaction within that research engagement action.	Using the research engagement action – searching for research, an example profile would be: a.ii) yes, research was searched for in academic literature databases (e.g., MEDLINE) or libraries; b.i) no, research was not searched for in sources of grey literature (e.g., OpenGREY); c.ii) yes, research was identified by chance or by colleagues; d.i) no, research was not identified by experts (researchers, working groups, librarians, or other research experts); e.ii) yes, research was searched for in search engines (e.g., Google) or social media sharing sites (e.g., Research Gate); f.i) no, reference lists, citation indices (e.g., Web of Science), or databases of references were not examined (e.g., EndNote).

### Developing a system to score research engagement actions

What is missing from the current scoring tool is a system that assigns a numeric score to each subaction and thus enables the calculation of a total score for each research engagement action. The calculation of a total score is useful because it can provide an indication of the extent to which policymakers have engaged with research in policy development. Numeric total scores can also be used to evaluate changes in policymakers’ engagement with research over time or following capacity-building interventions [32].

One obvious strategy for developing a scoring system would be to assign equal weights to each subaction. However, this would not be appropriate because certain subactions would arguably be considered stronger examples of engaging with research than others. For example, based on previous research and theory in evidence-informed policy, systematic reviews are considered to be a more informative resource than individual peer-reviewed studies [25,44]. Additionally, searching peer-reviewed literature databases is a more comprehensive search strategy than searching through Google [45]. Furthermore, although policymakers often appraise quality by examining the clarity and comprehensiveness of how the research was written up [40] or assessing the trustworthiness of the research producer [46], evidence indicates that a more thorough approach would be to evaluate the validity of the research methodology and conclusions [47,48].

Previous tools for assessing research engagement actions by policymakers, however, have not numerically quantified the relative importance of these subactions. One way to establish an appropriate numerical worth for each subaction is to seek the opinions of experts in health policy and research. Experts understand the policy context, and the constraints on using research in light of political influences, stakeholder interests, skill and resource limitations, and other contextual aspects. Consequently, they can provide informed and context-sensitive judgments regarding the relative importance of each research engagement subaction.

### Using conjoint analysis to develop a system to score research engagement actions

One systematic method of eliciting experts’ views regarding the value to assign to these subactions is conjoint analysis [49-53]. Conjoint analysis can be used to determine not only what products, services, or objects individuals prefer, but also what subactions [49] are the most important in driving these preferences [54]. In a typical conjoint analysis survey, respondents do not rate individual subactions^a^, but combinations of subactions, known as ‘profiles’ (see Table 1 for definitions). In the case of research engagement actions, it is more valid to evaluate profiles as opposed to individual subactions, because each research engagement action can be made up of a combination of subactions [19]. For example, a search for research will normally incorporate multiple subactions such as searching academic databases, using on-hand research, or examining reference lists. The conjoint analysis will allow identification of which subactions experts consider to be better or worse examples of each research engagement action by calculating numerical weights (called utilities) for each subaction. These utilities represent the score to be assigned to each subaction in the SAGE scoring tool.

We considered using other methods of obtaining expert opinion, such as verification and Delphi panels [55-58], however, these were not used mainly because they do not provide a systematic means of assigning numeric scores to individual subactions. Conjoint analysis, on the other hand, provides a systematic statistical method of assigning utilities (i.e., scores) to each subaction, thereby enabling the calculation of total scores for each research engagement action. Because of these advantages, conjoint analysis was used in the present study.

### Aim of the present study

The primary aim of the present study was to use conjoint analysis to develop an objective, structured, transparent, and context-sensitive system to score policymakers’ research engagement actions, based on the opinions and preferences of health policy and research experts.

## Methods

### Ethics

Ethics approval was granted by the University of Western Sydney Human Research Ethics Committee HREC Approval H10440. Written consent was obtained from all potential respondents prior to their participation in the study.

### Respondents

We specifically targeted individuals with experience working at the nexus between health policy and health research. Firstly, we identified relevant researchers who have investigated evidence-informed health policy by contacting the corresponding authors of key research articles in this area. Secondly, we contacted members in the CIPHER community to identify researchers and policymakers with experience in both health policy and health research. Using this method, 361 potential respondents were identified and invited by email to participate in the study.

### Procedure

We followed the guidelines specified by Bridges et al. [59] and Lancsar and Louviere [60] for designing, conducting, analysing, and reporting on the findings of choice experiments. Furthermore, we applied principles of Hierarchical Information Integration by identifying six separate research engagement actions (e.g., searching, appraising, generating research), identifying the key, non-overlapping subactions of each research engagement action, and undertaking a separate choice experiment for each action [61,62]. These steps are described below.

#### Defining the subactions and levels

We undertook a comprehensive, step-by-step approach to identify the subactions of each research engagement action. We first defined each of the six research engagement actions using the SPIRIT Action Framework [19], seminal research on knowledge translation, and Haynes and colleagues’ review of health policy definitions [63]. With these definitions in mind, we conducted a thorough analysis of the i) extant literature on evidence-informed health policymaking and ii) SAGE interviews with health policymakers, to identify a broad range of concrete and specific examples of each research engagement action. A vast number of examples of each research engagement action were identified. Similar examples were then categorised into groups. Each group was given an action label that encompassed all the examples within that group. These action labels became the subactions for a particular research engagement action. For example, hiring a consultant to locate research and contacting a researcher to identify relevant research were both examples of searching strategies identified in the literature (thus linked to the research engagement action: ‘Searching for research’). Both of these examples were grouped together to form a specific subaction of ‘Searching for research: using experts to find relevant research’.

Having identified the subactions of each research engagement action, the next step involved dividing each subaction into its ‘levels’ (Table 1). Levels in conjoint analysis refer to the possible values of a subaction [49]. Hair et al. [49] stress that levels should be stated in concrete terms. As a result we separated the majority of subactions into just two levels: i) Yes, the action was performed or ii) No, the action was not performed. Only one subaction contained more than two levels; this was the subaction representing the degree of intention expressed by the policymaker to generate new research. Identifying the levels of subactions was a necessary step before conducting the conjoint analysis, so that profiles could be created. Profiles are combinations of subaction levels (Table 1 and Additional file 2). The final list of subactions and their levels for each research engagement action is displayed in Table 2.

**Table 2:** **Research engagement actions, subactions, subaction levels, raw utilities, standard errors, and rescaled utility coefficients**

Research engagement action	Subaction	Levels of each subaction	Raw utility coefficient	Standard error	Rescaled utility coefficient†
1. Searching for research	a. Policymaker searched academic literature databases and/or physical libraries	i) No	−3.59***	0.27	0
		ii) Yes	0	–	2.83
	b. Policymaker searched grey literature sources	i) No	−1.81***	0.16	0
		ii) Yes	0	–	1.42
	c. Policymaker obtained research by chance, research that was on-hand, or provided by colleagues	i) No	−1.37***	0.11	0
		ii) Yes	0	–	1.08
	d. Policymaker requested experts (researchers, working groups, librarians, or other research experts) to identify research	i) No	−1.98***	0.16	0
		ii) Yes	0	–	1.56
	e. Policymaker searched generic databases or search engines	i) No	−1.12***	0.11	0
		ii) Yes	0	–	0.88
	f. Policymaker examined reference lists, citation indices, or databases of references	i) No	−1.55***	0.14	0
		ii) Yes	0	–	1.22
2. Research obtained and used	a. Policymaker found systematic reviews and/or meta-analyses	i) No	−1.94***	0.24	0
		ii) Yes	0	–	3.29
	b. Policymaker found books and/or technical monographs	i) No	−0.34***	0.12	0
		ii) Yes	0	–	0.58
	c. Policymaker found primary research and/or theoretical research	i) No	−0.99***	0.19	0
		ii) Yes	0	–	1.67
	d. Policymaker found unpublished research and/or conference resources	i) No	−0.49***	0.13	0
		ii) Yes	0	–	0.82
	e. Policymaker found internal policies, evaluations, or data	i) No	−0.23*	0.10	0
		ii) Yes	0	–	0.39
	f. Policymaker found policies, evaluations, or data from external organisations or registries	i) No	−0.60***	0.11	0
		ii) Yes	0	–	1.01
	g. Policymaker obtained recent (up-to-date) research from the above categories	i) No (Older research)	−0.73***	0.15	0
		ii) Yes (Recent)	0	–	1.24
3. Appraising relevance	a. Policymaker assessed whether the research was applicable to the policy context or policy issue	i) No	−1.70***	0.17	0
		ii) Yes	0	–	2.06
	b. Policymaker assessed whether research recommendations were actionable and/or feasible?	i) No	−1.53***	0.18	0
		ii) Yes	0	–	1.84
	c. Policymaker assessed whether the research was consistent with previous research on the issue	i) No	−1.02***	0.15	0
		ii) Yes	0	–	1.23
	d. Policymaker assessed if research was compatible with his/her OR the organisation’s values, knowledge, or experience	i) No	−0.97***	0.14	0
		ii) Yes	0	–	1.17
	e. Policymaker consulted experts to assess the relevance of research	i) No	−1.07***	0.12	0
		ii) Yes	0	–	1.29
	f. Policymaker undertook these actions as part of a pre-specified strategy	i) No (ad-hoc, unplanned)	−1.16***	0.18	0
		ii) Yes	0	–	1.40
4. Appraising quality	a. Policymaker assessed whether the design or conclusions of the research were valid	i) No	−1.16***	0.22	0
		ii)Yes	0	–	2.00
	b. Policymaker evaluated whether the design or conclusions of the research were described clearly and comprehensively	i) No	−0.68***	0.16	0
		ii) Yes	0	–	1.17
	c. Policymaker assessed whether the source of the research was credible	i) No	−0.64***	0.12	0
		ii) Yes	0	–	1.10
	d. Checked if the research cited, or was referenced in other high-quality research or policy documents	i) No	−0.45**	0.18	0
		ii) Yes	0	–	0.77
	e. Policymaker consulted experts to assess quality	i) No	−0.76***	0.19	0
		ii) Yes	0	–	1.31
	f. Policymaker assessed the level of evidence of the research	i) No	−0.88***	0.17	0
		ii) Yes	0	–	1.51
	g. Policymaker undertook these actions as part of a pre-specified strategy	i) No (ad-hoc, unplanned)	−0.67***	0.22	0
		ii) Yes	0	–	1.15
5. Generating new researchers	a. Policymaker expressed explicit intentions to generate or commission new research (to follow-up the current policy) OR stated that he/she had already undertaken this research	i) No (no intentions to generate new research)	−2.08***	0.28	0
		ii) No (uncertain intentions only)	−1.97***	0.27	0.18
		iii) Yes	0	–	3.42
	b. Policymaker mentioned thorough research generation activities	i) No	−1.72***	0.19	0
		ii) Yes	0	–	2.84
	c. Policymaker mentioned less intensive research activities	i) No	−0.96***	0.14	0
		ii) Yes	0	–	1.58
	d. Policymaker advocated for future research to be undertaken	i) No	−0.60***	0.15	0
		ii) Yes	0	–	0.99
6. Interacting with researchers	a. Policymaker engaged in thorough collaborative activities with researchers	i) No	−2.56***	0.25	0
		ii) Yes	0	–	3.75
	a. Policymaker engaged in less intensive interactions with (other) researchers	i) No	−0.91***	0.11	0
		ii) Yes	0	–	1.33
	b. Policymaker engaged in sporadic contact with (other) researchers?	i) No	−0.67***	0.11	0
		ii) Yes	0	–	0.98
	c. Policymaker actively initiated these interaction activities	i) No	−2.01***	0.22	0
		ii) Yes	0	–	2.94

^†^Utility coefficients were rescaled so that they became positive, with the lowest level of each subaction having a zero-coefficient, and adding up to 9.

^***^p < 0.001; ^**^p < 0.01; ^*^p < 0.05.

#### The experimental design

The full profile method was used [49] where each profile consisted of combinations of levels across all subactions. The subactions and levels gave rise to a large number of possible profiles, particularly for the following research engagement actions: ‘Searching for research’, ‘Research obtained’, ‘Appraising relevance’, and ‘Appraising quality’*.* The number of profiles for each research engagement action was reduced to a manageable number (i.e., eight profiles) using an Orthogonal Main Effects Plan (OMEP) in R software [50]. The OMEP generated a series of orthogonal and balanced profiles for each of the six conjoint analyses. This was appropriate because we were only interested in main effects (i.e., the utility values assigned to each research engagement subaction level) and not interactions among subaction levels [49]. An OMEP was also advantageous because it generated only eight profiles for each research engagement action, which would enhance the efficiency of the task and reduce the cognitive load for our sample (see Additional file 2 for the complete list of profiles).

#### Eliciting preferences

**Figure 2:**
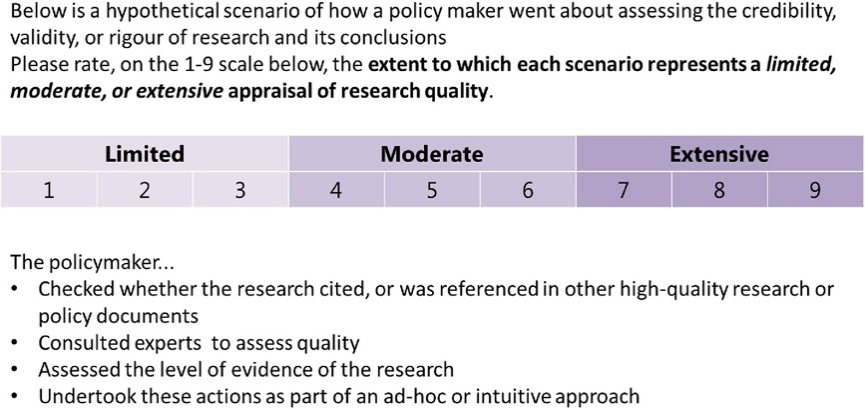
Example scenario for conceptual research use.

In order to elicit respondents’ preferences, they were instructed to rate the standard of each profile on the same 1 to 9 ordinal scale (Figure 2). Profiles were presented using online surveys created with Survey Monkey software [64]. Respondents completed at most six surveys, one for each research engagement action. All the surveys were housed within the one Survey Monkey domain (https://www.surveymonkey.com/s/SAGE_Conjoint) and completed within the same session. The survey order was as follows: ‘Searching for research’, ‘Research obtained’, ‘Appraising relevance’, ‘Appraising quality’, ‘Generating New research or analyses’, and ‘Interacting with researchers’.

All potential respondents were contacted by email, with study information and a link to the online survey provided therein. The first page of the online survey was an online consent form. On the second page, respondents entered personal details, including their assigned ID number (which was sent with their invitation email), affiliation, and current working role, which they could select as either ‘policymaker’, ‘researcher’, or ‘other’. If ‘other’ was chosen, they were required to specify their working role in a textbox. After providing their details, respondents were presented with the six conjoint surveys for each research engagement action. Each survey with its corresponding profiles was presented on a separate page. See Figure 2 for an example of a profile respondents rated for ‘Research obtained’. All respondents were presented with the same set of eight orthogonal profiles generated from the OMEP. Respondents were required to rate, on a 1 to 9 scale, whether the profile represented a limited (1–3), moderate (4–6), or an extensive (7–9) form of the research engagement action in question. The presentation order of profiles was randomised across respondents. Respondents were required to rate all eight profiles for a particular research engagement action before moving onto the next page.

#### Data analyses

The data was analysed using generalised estimating equations (GEE), which is appropriate when ratings are made on an ordinal scale, predictors are categorical, and participants provide multiple responses [50,65-67]. A link function and working correlation structure are to be specified when using GEE procedures [66]. Alternative procedures were considered, such as multilevel modelling, which also accounts for repeated observations, although most statistical software packages do not have provisions for ordinal multilevel analysis [68]. Fixed effects models were not appropriate because observations were correlated within subjects (see below).

Six conjoint analyses were undertaken using the GEE procedure in SPSS (SPSS GENLIN) with a logit link function and robust estimator. An exchangeable working correlation structure was used because examination of the within-subject correlation structure revealed that ratings of profiles within subjects were correlated at a relatively similar magnitude [69]. There were problems with convergence due to singularity on all conjoint analyses (except ‘Searching for research’ and ‘Generating new research’). Singularity occurs when independent variables (i.e., the subactions) are perfectly correlated such that one variable is a perfect combination of one or more other variables, and often arises due to small sample sizes relative to the number of variables [69]. Singularity can lead to unreliable estimates of utilities because it blocks the iterative process [70]. Therefore, based on the recommendations of Lipsitz et al. [71], the one-step estimator (i.e., utility estimates obtained from one iteration of the GEE) was used, since evidence demonstrates that the estimates obtained are similar to fully iterated GEE results [69]. To verify the findings, a fixed effects model was also estimated by setting the working correlation structure to ‘independent’ in SPSS GENLIN, and this yielded similar utility estimates (results not reported).

Raw regression coefficients for each subaction level were calculated, which represented the part-worth utilities of each subaction level. To make the part-worth utilities meaningful, they were rescaled into a positive value out of 9 using the guidelines provided by Hair et al. [49]. Larger rescaled utility values indicated that a certain subaction level was particularly influential in guiding respondents’ ratings. Importance values were calculated using equation (1) below, based on the guidelines of Hair et al. [49] to quantify the relative importance of each subaction. Larger importance values indicate that a particular subaction was influential in guiding respondents’ ratings.

1 $$ Importance\; of\; attribute=\frac{\left({\mathrm{range}}^{\dagger}\;\mathrm{o}\mathrm{f}\;\mathrm{utilities}\;\mathrm{f}\mathrm{o}\mathrm{r}\;\mathrm{s}\mathrm{pecified}\;\mathrm{s}\mathrm{ubaction}\right)}{\left(\mathrm{sum}\;\mathrm{o}\mathrm{f}\;\mathrm{the}\;\mathrm{r}\mathrm{ange}\;\mathrm{o}\mathrm{f}\;\mathrm{utilities}\kern0.5em \mathrm{across}\;\mathrm{all}\;\mathrm{s}\mathrm{ubaction}\mathrm{s}\right)}\times 100 $$

† The range is the largest utility minus the smallest utility within a subaction.

SPSS Conjoint was used to identify respondents that exhibited ‘reversals’ *–* highly inconsistent responses and illogical patterns in preferences for particular subaction levels [49]. Hair et al. [49] proposed that respondents who display many reversals are potential candidates for deletion from the analyses.

## Results

### Respondent characteristics

Out of the 361 participants invited, 93 consented and 69 respondents (19.1%) completed at least the first conjoint analysis (Searching for research). A total of 60 respondents completed ‘Research obtained’, 56 respondents completed ‘Appraising relevance’, 55 completed ‘Appraising quality’ and ‘Generating new research’, and 53 completed ‘Interacting with researchers’. Thus, at least 53 respondents (14.5%) completed all six surveys. Based on Orme’s [72] guidelines regarding the appropriate sample size for investigational work and developing hypotheses about a particular group (i.e., between 30 and 60), our sample size was sufficient.

Respondent characteristics are displayed in Table 3 for the sample that completed at least the first conjoint analysis (*n =* 69). There were significantly more females than males (χ^2^ (2, *n* = 69) = 5.23, *p* = 0.02). There were no significant differences in the number of respondents between each working role group (χ^2^ (3, *n* = 69) = 3.64, *p* = 0.30).

**Table 3:** **Respondent characteristics**

			Working role				Total
			Policymaker	Researcher	Both researcher and policymaker	Other	
Sex	Male	Count	6	9	6	4	25
		% of total	8.7%	13.0%	8.7%	5.8%	36.2%
	Female	Count	10	15	11	8	44
		% of total	14.5%	21.7%	15.9%	11.6%	63.8%
Total	Count	16	24	17	12	69	
	% of total	23.2%	34.8%	24.6%	17.4%	100.0%	

### Conjoint analysis findings for each research engagement action

For each research engagement action, we display the raw and rescaled part-worth utilities in Table 2, and the importance values in Table 4. The rescaled part-worth utilities represent the score assigned to each subaction in the SAGE scoring tool.

**Table 4:** **Research engagement action subactions and their importance values**

Research engagement action	Subaction	Importance† (%)
1. Searching for research	a. Policymaker searched academic literature databases and/or physical libraries	31.45
	b. Policymaker searched grey literature sources	15.82
	c. Policymaker obtained research by chance, research that was on-hand, or provided by colleagues	12.04
	d. Policymaker requested experts (researchers, working groups, librarians, or other research experts) to identify research	17.32
	e. Policymaker searched generic databases or search engines	9.80
	f. Policymaker examined reference lists, citation indices, or databases of references	13.56
2. Research obtained	a. Policymaker found systematic reviews and/or meta-analyses	36.50
	b. Policymaker found books and/or technical monographs	6.42
	c. Policymaker found primary research and/or theoretical research	18.61
	d. Policymaker found unpublished research and/or conference resources	9.16
	e. Policymaker found internal policies, evaluations, or data	4.28
	f. Policymaker found policies, evaluations, or data from external organisations or registries	11.27
	g. Policymaker obtained recent (up-to-date) research from the above categories	13.75
3. Appraising relevance	a. Policymaker assessed whether the research was applicable to the policy context or policy issue	22.84
	b. Policymaker assessed whether research recommendations were actionable and/or feasible	20.50
	c. Policymaker assessed whether the research was consistent with previous research on the issue	13.70
	d. Policymaker assessed if research was compatible with his/her OR the organisation's values, knowledge, or experience	13.05
	e. Policymaker consulted experts to assess the relevance of research	14.35
	f. Policymaker undertook these actions as part of a pre-specified strategy	15.56
4. Appraising quality	a. Policymaker assessed whether the design or conclusions of the research were valid	22.17
	b. Policymaker evaluated whether the design or conclusions of the research were described clearly and comprehensively	12.98
	c. Policymaker assessed whether the source of the research was credible	12.17
	d. Checked if the research cited, or was referenced in other high-quality research or policy documents	8.60
	e. Policymaker consulted experts to assess quality	14.51
	f. Policymaker assessed the level of evidence of the research	16.77
	g. Policymaker undertook these actions as part of a pre-specified strategy	12.79
5. Generating new researcher	a. The level of intention of the policymaker to generate or commission new research (to follow-up the current policy)	38.79
	b. Policymaker mentioned thorough research generation activities	32.17
	c. Policymaker mentioned less intensive research activities	17.85
	d. Policymaker advocated for future research to be undertaken	11.19
6. Interacting with researchers	a. Policymaker engaged in thorough collaborative activities with researchers	41.78
	b. Policymaker engaged in less intensive interactions with (other) researchers	14.09
	c. Policymaker engaged in sporadic contact with (other) researchers?	11.03
	d. Policymaker actively initiated these interaction activities	33.10

† Importance values were calculated by dividing a subaction’s range (i.e., highest utility minus the lowest utility) by the sum of ranges across all subactions.

#### Searching for research

Two respondents exhibited five or more reversals (i.e., highly inconsistent responses for five or more of the subactions) and were eliminated from the analyses (see Method). Sixty seven respondents were included in the analyses. All raw utility coefficients were significant and negative, implying that performing each of the six key actions, on their own, was related to more positive ratings. Based on the rescaled coefficients and importance values, the most important subaction that influenced respondents’ ratings of searching for research was using academic literature databases, followed by consulting experts, searching grey literature sources, and examining reference lists and citations. Using on-hand research and generic search engines were the least important subactions, but were nonetheless significantly related to higher ratings.

#### Research obtained and used

One respondent gave equal ratings on all profiles, whereas two others exhibited five or more reversals. These respondents were eliminated from the analyses. An additional four respondents dropped out of the study. Sixty respondents were included in the analyses. Each raw utility coefficient was significant and negative, implying that obtaining each of the six resources was, on its own, related to higher ratings for this particular research engagement action. If we apply a more stringent significance level (i.e., 0.05/7 = 0.007), ‘internal policies, evaluations, or data’ was not a significant subaction. Based on the rescaled coefficients and importance values, respondents overwhelmingly placed the greatest value on obtaining systematic reviews. This was followed by peer-reviewed research (whose importance value was half that of systematic reviews), policies/evaluations/data from external organisations, and unpublished research or conference resources. Respondents placed the least importance on obtaining internal policies/evaluations/data or books/technical monographs. Finally, respondents valued the use of recent as opposed to older research.

#### Appraising the relevance of research

One respondent gave equivalent ratings for all profiles, and one respondent exhibited six reversals. Both were eliminated from the analyses. One additional respondent dropped out of the study, which left 57 respondents in the analyses. Each raw utility coefficient was significant and negative, implying that each of the six key actions was, on its own, related to more positive ratings of appraising relevance. Based on the rescaled coefficients and importance values, respondents regarded the most important aspects of appraising relevance as assessing whether research is applicable to the policy context and/or issue; and assessing whether the recommendations in the research are actionable and/or feasible. The other three aspects (i.e., requesting experts to appraise the relevance of research; assessing if research is compatible with the knowledge, values, or experience of the policymaker/organisation, and evaluating whether research is consistent with previous research) had similar-sized regression coefficients, indicating comparable impact on respondents’ ratings. Finally, respondents valued policymakers undertaking relevance appraisal as part of a pre-specified plan as opposed to an ad-hoc strategy.

#### Appraising the quality of research

Two respondents exhibited reversals on six of the key aspects of quality appraisal, and these were eliminated, which left 55 respondents included in the analyses. All raw utility coefficients were significant and negative, implying that each action was positively related to ratings of relevance appraisal. However, when applying a more stringent significance level (0.05/7 = 0.007), ‘checking if research was cited in, or cited other high quality research’ was not a significant subaction. From the rescaled coefficients and importance values, the three subactions that respondents regarded as most important to assessing quality were assessing whether the design or conclusions of the research study were valid, assessing the level of evidence of the research, and requesting experts to assess relevance. This was followed by evaluating the comprehensibility of the design and conclusions, and assessing the credibility of the source of the research. Respondents placed the least importance on examining whether the research cited, or was cited in, other high quality research studies. Finally, respondents valued policymakers undertaking quality appraisal as part of a pre-specified plan as opposed to an ad-hoc strategy.

#### Generating new research and/or analyses

One respondent displayed reversals on all four key aspects of generating new research, which left 54 respondents in the analyses. All raw utilities were significant and negative, indicating that each aspect was positively related to higher ratings of quality appraisal. The most important subaction was the degree of intention expressed by the policymaker to generate new research. Based on the rescaled utilities, respondents greatly valued policymakers having definite and explicit intentions to generate new research (as opposed to having uncertain or no intentions). In terms of specific research generation activities, respondents placed a great deal of importance on policymakers undertaking thorough research generation activities. The importance value for this subaction was almost double that of undertaking less intensive interactions, and almost triple that of advocating for new research to be undertaken.

#### Interacting with researchers

One respondent displayed reversals on all four key aspects of generating new research, which left 53 respondents in the analyses. All raw utilities were significant and negative, indicating that each aspect was positively related to higher ratings of interacting with researchers. The rescaled part-worth and importance values indicate that respondents greatly valued policymakers deliberately initiating interactions with researchers. In terms of the specific kinds of research activities, respondents most valued when policymakers undertook thorough collaborative activities with researchers. The importance of this subaction was more than three times that of engaging in less intensive interactions, or engaging in sporadic contact with researchers.

#### Developing a scoring system to assess research engagement actions

**Figure 3:**
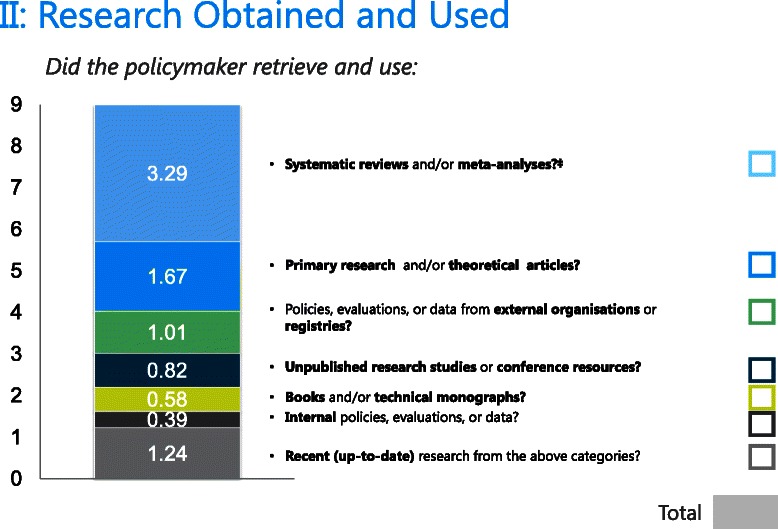
Scoring tool for research obtained.

The (rescaled) utilities can be used to score each research engagement action in the SAGE scoring tool. These utilities represent the numerical score assigned to that particular subaction in the tool. If a policymaker reports having engaged in a particular subaction, the score is assigned for that subaction. Using research obtained as an example, if a policymaker reports in the SAGE interview that he/she obtained systematic reviews (utility = 3.75) and primary research (utility = 1.77), and both resources were recent (utility = 1.30), he/she would be assigned a score of 3.75 + 1.77 + 1.30 = 6.82, which rounded up would represent a score of 7 out of 9, indicating extensive research obtained (Figure 3). The full scoring tool is provided in Additional file 3.

## Discussion

The use of conjoint analysis with a sample of experts working at the health research and policy nexus provided us with a systematic and innovative method to quantify the relative importance of subactions for each research engagement action in the SAGE tool. While, to our knowledge, the current study represents the first quantitative attempt to describe the relative importance of different research engagement action subactions, our findings are highly consistent with previous qualitative research in this area as will be discussed below. This increases our confidence that the conjoint analysis extracted valid utility values for each subaction, and points to the face validity and appropriateness of our scoring system. In the discussion that follows we firstly summarise and explore the present findings in relation to previous research. We then discuss the advantages of the SAGE scoring system particularly in relation to evidence-informed policymaking. Finally, we outline some of the limitations of the present study and the implications for future research.

### Summary and exploration of findings in relation to previous research

For ‘Searching for research’, the most important subaction in guiding experts’ choices was searching academic literature or systematic review databases. This result makes sense, given that searching academic or systematic review databases will most likely allow policymakers to obtain the most relevant, thorough, and up-to-date research on a particular health issue [23,44,73]. Similarly, it’s unsurprising that low utility values emerged for subactions such as using on-hand research or searching generic search engines like Google, as such methods may not identify the breadth of relevant research nor allow users to access these resources directly [74].

For ‘Research obtained’, systematic reviews overwhelmingly yielded the highest overall utility value and importance. Systematic reviews are valuable because they can provide robust information about the level of evidence for the effect of interventions, help policymakers clarify policy problems, aid in the identification of possible strategies to address these problems, and highlight a number of considerations for implementing them, including costs, resources, and other constraints [44]. Respondents also valued policymakers obtaining primary research and theoretical articles, although this was judged to be less than half as important as accessing systematic reviews. Indeed, Whitehead et al. [75] found that policymakers highly valued particular kinds of primary research, including observational and household studies, controlled intervention evaluations, or historical studies with long shelf life. Interestingly, obtaining internal policies/evaluations/data did not yield a high utility value, nor was this subaction significantly related to choices, which may be reflective of the growing importance of utilising ‘citable’, as opposed to internally produced, research [76]. Indeed, evidence indicates that policymakers want to use robust information (e.g., from systematic reviews) to inform decision making, but often prioritise locally relevant information sources due to limited resources and time, and perceived deficits in research skills [23].

The subactions with the greatest importance to ‘Appraising relevance’ of research were assessing whether the research was applicable to the policy context or issue, and assessing if recommendations and strategies were actionable or feasible within the current context. This result aligns with previous studies documenting the importance of research recommendations corresponding with the local setting, addressing the needs and values of targeted stakeholders, and conveying a clear and direct course of action that can be performed within current resource constraints [15,29,36,40,43].

For ‘Appraising quality’, the highest utility estimates were for assessing the validity of the design or conclusions, and assessing the level of evidence of the research. This indicates that our expert respondents placed the greatest importance on policymakers evaluating the validity and methodological quality of the design of a study, and ensuring that conclusions were appropriate, at low risk of bias, and correct. These two subactions align with previous research with policymakers stressing the importance of research being valid, at low risk of bias, and high in technical quality and statistical sophistication [3,36,40,77,78].

For ‘Generating new research’ and ‘Interacting with researchers’, respondents placed great value on policymakers actively initiating thorough research generation activities and/or collaborations with researchers (i.e., formalised research projects where findings are documented, analysed, and reported, often with active and sustained involvement of both policymakers and researchers). The importance value for this subaction was almost double that of less intensive research generation activities (e.g., informal data gathering through activities such as workshops or working groups) and more than triple that of engaging in less intensive interactions (i.e., interactions with researchers that are direct but unsustained such as one-off forums, formal meetings, or seminars). These findings agree with previous studies emphasising the value and importance of policymakers being ‘integral research partners’, where they deliberately seek out collaborations with researchers, and are actively involved in all key stages of the research project (i.e., research design, data collection, translation, and use in policy) [25,26]. Indeed, Innvaer et al.’s [28] systematic review revealed that personal contact between researchers and policymakers was one of the most commonly mentioned facilitators of research use by policymakers. The utility values which emerged are consistent with these findings.

### Advantages of the SAGE scoring system and implications for evidence-informed policymaking

A key strength of SAGE as a measuring tool is the empirically derived scoring system generated in the present study. We have used conjoint analysis for the first time to calculate utilities that quantify the relative importance of key research engagement subactions, based on the opinions and preferences of experts in health policy and research. We have then used these utilities to generate a context-appropriate and valid system of scoring policymakers’ engagement with research in the development of policy.

Using our empirically validated scoring tool, policy organisations can now use SAGE to measure their current research engagement actions, identify the most useful targets for increasing their research engagement capacity, and track changes in their research engagement actions over time. For example, an organisation may initially use SAGE to evaluate the extent to which staff currently interacts with researchers to inform policy development. They would find, however, that they would score very low on this research engagement action. The scoring tool shows that the subaction with the highest utility is 6a: engaging in thorough collaborative activities with researchers (e.g., collaborating with researchers to design, conduct, and/or analyse the results of a policy-relevant research project). Consequently, the organisation could decide to invest in structures that facilitate the formation of collaborative partnerships with researchers (e.g., they develop clear guidelines on how to commission researchers to undertake a research project), so that priority research projects can be conducted to inform policy [21,26]. The organisation could then readminister SAGE to evaluate whether these structures have successfully improved the extent and quality of staff interactions with researchers during policy development. Evidence shows that improvements in policymakers’ interactions with researchers will likely increase the extent to which research informs the organisation’s policies and programs [26,79], ultimately maximising the long-term health benefits and cost-savings of their policies [2].

Consistent with the above, SAGE is currently being used as the primary outcome measure in SPIRIT, an organisation-wide study examining the impact of a multifaceted program designed to improve the capacity of policy agencies and staff to engage with and use research in policy and program development [32]. SAGE will be used to evaluate whether the program has improved the extent to which policymakers have searched for, accessed, appraised, and generated research, and interacted with researchers during the development of policy documents.

### Limitations and implications for future research

A possible limitation of the current study is that the subactions included in the conjoint analysis (and thus, in the scoring tool itself) did not capture the complete breadth and diversity of each research engagement action. This is unlikely, however, since the subactions were obtained by categorising a vast range of examples of each research engagement action identified from the literature on evidence-informed policymaking and interviews with policymakers.

We opted not to include an excessive number of subactions in order to reduce the complexity of the conjoint task. We must acknowledge, however, that based on survey comments, some respondents experienced difficulties doing the survey due to response burden. This may have occurred because, firstly, respondents were required to complete six conjoint surveys. Secondly, all profiles for a particular research engagement action were presented on a single page and the subactions were not one-word physical descriptions as is typically used in choice studies. Therefore, the task was quite involved. If a conjoint task is too complex, then respondents are less likely to be engaged, leading to deficits in the reliability of ratings across profiles or a lack of differentiation between profiles [49]. Indeed, as highlighted in the results section, a small number of respondents exhibited reversals in each conjoint task, while others provided identical ratings across all profiles. Nonetheless, as discussed above, the utility estimates we obtained were consistent with previous research and theory regarding the subactions policymakers and researchers value most when engaging with research. This increases our confidence that participants were effectively able to engage with the conjoint task, and that the obtained utility values for each subaction were valid.

Another limitation relates to the fairly low response rate achieved. Consequently, we may have recruited a non-representative sample of health policy experts. However, as our final sample consisted of a range of senior researchers and health policymakers from local and international organisations, we are confident that we recruited reasonably knowledgeable and authoritative experts in this area. Nonetheless, future studies should include experts from developing countries, as they may have different perspectives regarding which subactions are the most important for fulfilling each research engagement action. SAGE would also benefit from further research regarding its construct validity. We are currently undertaking studies to further test the reliability and validity of the SAGE scoring tool.

## Conclusions

In this study, we have used conjoint analysis with a sample of experts in health policy and research, to generate a novel, informative, and context-sensitive system to score the research engagement actions undertaken by policymakers during the development of a health policy or program document. This scoring system breaks down each research engagement action into its key subactions. Points are assigned to each subaction based on experts’ opinions regarding which subactions are most important to effectively engage with research to inform policy development. This empirically derived scoring system will not only allow policy organisations to quantify the research engagement actions undertaken by staff, but also help them identify the most useful targets for increasing their research engagement capacity, which could ultimately lead to improvements in the development of evidence-informed policies.

## Endnotes

^a^In a typical conjoint analysis, the subactions would be referred to as ‘attributes’ [49]; however, we used the term subactions to enhance clarity and consistency of terms throughout this paper.

## Additional files

Additional file 1:
**SAGE: Assessing the use of research in policy products - Interviewer’s guide.**
Additional file 2:
**Profiles and questions in the SAGE ONLINE survey.**
Additional file 3:
**SAGE: Scoring tool.**

## Abbreviations

CIPHER: Centre for Informing Policy in Health with Evidence from Research
GEE: Generalised estimating equations
OMEP: Orthogonal Main Effects Plan
SAGE: Staff Assessment of enGagement with Evidence from research
SPIRIT: Supporting Policy in Health with Research: an Intervention Trial
SPSS GENLIN: GEE procedure in SPSS

## References

[1] Black N (2001). Evidence based policy: proceed with care. Brit Med J.

[2] Brownson RC, Chriqui JF, Stamatakis KA (2009). Understanding evidence-based public health policy. Am J Public Health.

[3] Brownson RC, Fielding JE, Maylan CM (2009). Evidence-based public health: a fundamental concept for public health practice. Annu Rev Public Health..

[4] Hansen J (2011). Health services research in Europe: evaluating and improving its contribution to health care policy. J Health Serv Res Policy.

[5] Lavis J (2006). Research, public policymaking, and knowledge-translation processes: Canadian efforts to build bridges. J Contin Educ Heal Prof..

[6] Buchan H (2004). Gaps between best evidence and practice: causes for concern. Med J Australia..

[7] Fielding JE, Briss PA (2006). Promoting evidence-based public health policy: can we have better evidence and more action?. Health Aff (Millwood)..

[8] Andre FE, Booy R, Bock HL, Clemens J, Datta SK, John TJ (2008). Vaccination greatly reduces disease, disability, death and inequity worldwide. Bull World Health Organ.

[9] Hanna JN, Hills SL, Humphreys JL (2004). Impact of hepatitis A vaccination of Indigenous children on notifications of hepatitis A in north Queensland. Med J Aust.

[10] Morrato EH, Elias M, Gericke CA (2007). Using population-based routine data for evidence-based health policy decisions: lessons from three examples of setting and evaluating national health policy in Australia, the UK and the USA. J Public Health.

[11] Elshaug AG, Hiller JE, Tunis SR, Moss JR (2007). Challenged in Australian policy processes for disinvestment from existing, ineffective health care practices. Aust New Zealand Health Policy..

[12] Ham C, Hunter DJ, Robinson R (1995). Evidence based policymaking: research must inform health policy as well as medical care. Brit Med J..

[13] Orton L, Lloyd-Williams F, Taylor-Robinson D, O’Flaherty M, Capewell S (2011). The use of research evidence in public health decision making processes: systematic review. PLoS One.

[14] Weiss C (1980). Knowledge creep and decision accretion. Sci Commun.

[15] Amara N, Ouimet M, Landry R (2004). New evidence on instrumental, conceptual, and symbolic utilization of university research in government agencies. Sci Commun.

[16] Campbell DM, Redman S, Jorm L, Cooke M, Zwi AB, Rychetnik L (2009). Increasing the use of evidence in health policy: practice and views of policy makers and researchers. Aust New Zealand Health Policy..

[17] Chagnon F, Poullot L, Malo C, Gervais MJ, Pigeon ME (2010). Comparison of determinants of research knowledge utilization by practitioners and administrators in the field of child and family social services. Implement Sci..

[18] El-Jardali F, Lavis JN, Ataya N, Jamal D (2012). Use of health systems and policy research evidence in the health policymaking in eastern Mediterranean countries: views and practices of researchers. Implement Sci..

[19] Redman S, Turner T, Davies H, Haynes A, Williamson A, Milat A, et al. on behalf of the CIPHER Team. The SPIRIT Action Framework: A structured approach to selecting and testing strategies to increase the use of research in policy. Soc Sci Med. Ahead of print. 10.1016/j.socscimed.2015.05.009 26004208

[20] Ritter A (2009). How do drug policy makers access research evidence?. Int J Drug Policy..

[21] Ellen ME, Lavis JN, Ouimet M, Grimshaw J, Bedard PO (2011). Determining research knowledge infrastructure for healthcare systems: a qualitative study. Implement Sci..

[22] Ettelt S, Mays N (2011). Health services research in Europe and its use for informing policy. J Health Serv Res Policy.

[23] Evans BA, Snooks H, Howson H, Davies M (2013). How hard can it be to include research evidence and evaluation in local health policy implementation? Results from a mixed methods study. Implement Sci..

[24] Hyder AA, Corluka A, Winch PJ, El-Shinnawy A, Ghassany H, Malekafzali H (2011). National policymakers speak out: are researchers giving them what they need?. Health Policy Plan..

[25] Lavis JN, Davies H, Oxman A, Denis JL, Golden-Biddle K, Ferlie E. Towards systematic reviews that inform health care management and policy-making. J Health Serv Res Policy. 2005;10(Suppl 1):S1:35–48. 10.1258/1355819054308549 16053582

[26] Mirzoev TN, Omar MA, Green AT, Bird PK, Lund C, Ofori-Atta A (2012). Research-policy partnerships – experiences of the Mental Health and Poverty Project in Ghana, South Africa, Uganda and Zambia. Health Res Policy Syst..

[27] Petticrew M, Whitehead M, Macintyre SJ, Graham H, Egan M (2004). Evidence for public health policy on inequalities: 1: the reality according to policymakers. J Epidemiol Community Health.

[28] Innvaer S, Vist G, Trommald M, Oxman A (2002). Health policy-makers’ perceptions of their use of evidence: a systematic review. J Health Serv Res Policy.

[29] Ssengooba F, Atuyambe L, Kiwanuka SN, Puvanachandra P, Glass N, Hyder AA (2011). Research translation to inform national health policies: learning from multiple perspectives in Uganda. BMC Inter Health Human Rights.

[30] Oliver K, Innvar S, Lorenc T, Woodman J, Thomas J (2014). A systematic review of barriers to and facilitators of the use of evidence by policymakers. BMC Health Serv Res..

[31] Helmsey-Brown J (2004). Facilitating research utilisation: a cross-sector review of research evidence. Inter J Pub Sector Manag.

[32] CIPHER Investigators (2014). Supporting Policy. In Health with Research: an Intervention Trial (SPIRIT)-protocol for a stepped wedge trial. BMJ Open.

[33] Squires JE, Estabrooks CA, O’Rourke HM, Gustavsson P, Newburn-Cook CV, Wallin L (2011). A systematic review of the psychometric properties of self-report research utilization measures used in healthcare. Implement Sci.

[34] de Goede J, van Bon-Martens MJ, Putters K, Van Oers HA (2012). Looking for interaction: quantitative measurement of research utilization by Dutch local health officials. Health Res Policy Syst..

[35] Zardo P, Collie A (2014). Measuring use of research evidence in public health policy: a policy content analysis. Implement Sci..

[36] Weiss CH, Bucuvalas MJ (1980). Truth tests and utility tests: decision-makers’ frames of reference for social science research. Am Sociol Rev.

[37] Sumner A, Crichton J, Theobald S, Zulu E, Parkhurst J. What shapes research impact on policy? Understanding research uptake in sexual and reproductive health policy processes in resource poor contexts. Health Res Policy Syst. 2011;(9 Suppl)1:53. 10.1186/1478-4505-9-S1-S3 PMC3121134 21679384

[38] Weiss CH (1979). The many meanings of research utilization. Public Adm Rev.

[39] Squires JE, Estabrooks CA, Newburn-Cook CV, Gierl M (2011). Validation of the conceptual research utilization scale: an application of the standards for educational and psychological testing in healthcare. BMC Health Serv Res..

[40] Weiss C, Bucuvalas MJ (1980). Social science research and decision-making.

[41] Beyer JM (1997). Research utilisation: bridging a gap between communities. J Manage Inquiry..

[42] Liverani M, Hawkins B, Parkhurst JO (2013). Political and institutional influences on the use of evidence in public health policy. PLoS One.

[43] Beyer JM, Trice HM (1982). The utilization process: a conceptual framework and synthesis of empirical findings. Adm Sci Q.

[44] Moat KA, Lavis JN, Wilson MG, Rottingen JA, Barninghausen T (2013). Twelve myths about systematic reviews for health system policymaking rebutted. J Health Serv Res Policy..

[45] Lavis JN, Robertson D, Woodside JM, McLeod CB, Abelson J (2003). How can research organizations more effectively transfer research knowledge to decision makers?. Milbank Q.

[46] Schur CL, Berk ML, Silver LE, Yegian JM, O’Grady MJ (2009). Connecting the Ivory Tower to Main Street: setting research priorities for real-world impact. Health Aff (Millwood).

[47] Lewin S, Oxman AD, Lavis JN, Fretheim A (2009). SUPPORT Tools for evidence-informed health policymaking (STP) 8: Deciding how much confidence to place in a systematic review. Health Res Policy Syst.

[48] Oxman A, CooK DJ, Guyatt GH (1994). Users’ guides to the medical literature. VI. How to use an overview. Evidence-Based Medicine Working Group. JAMA..

[49] Hair JF, Black WC, Babin BJ, Anderson RE, Tatham RL (2006). Multivariate data analysis: Sixth Edition.

[50] Bak A, Bartlomowicz T (2009). Conjoint analysis method and its implementation in conjoint R package.

[51] Ryan M (1999). Using conjoint analysis to take account of patient preferences and go beyond health outcomes: an application to in vitro fertilisation. Soc Sci Med..

[52] Carson RT, Louviere J (2011). A common nomenclature for stated preference elicitation approaches. Environ Resource Econ..

[53] Louviere J, Flynn TN, Carson RT (2010). Discrete choice experiments are not conjoint analysis. J Choice Modelling.

[54] Ryan M, Farrar S (2000). Using conjoint analysis to elicit preferences for health care. Brit Med J..

[55] Brook RH, Chassin MR, Fink A, Solomon DH, Kosecoff J, Park RE (1986). A method for the detailed assessment of the appropriateness of medical technologies. Int J Technol Assess Health Care.

[56] Hsu CC, Sanford BA (2007). The Delphi technique: making sense of consensus. Practical Assess, Res & Eval.

[57] Shekelle P (2004). The appropriateness method. Med Decis Making..

[58] Wortman PM, Smyth JM, Langenbrunner JC, Yeaton WH (1998). Consensus among experts and research synthesis: a comparison of methods. Int J Technol Assess Health Care.

[59] Bridges JF, Hauber AB, Marshall D, Lloyd A, Prosser LA, Regier DA (2011). Conjoint analysis applications in health - a checklist: a report of the ISPOR Good Research Practices for Conjoint Analysis Task Force. Value Health.

[60] Lancsar E, Louviere J (2008). Conducting discrete choice experiments to inform healthcare decision making: a user's guide. Pharmacoeconomics.

[61] Oppewal H, Louviere JJ, Timmermans HJP (1994). Modeling hierarchical conjoint processes with integrated choice experiments. J Mark Res.

[62] Louviere J (1984). Hiearchical information integration: a new method for the design and analysis of complex multiattribute judgment problems. Adv Consum Res..

[63] Haynes A, Turner T, Redman S, Milat A, Moore G (2015). Developing definitions for a knowledge exchange intervention in health policy and program agencies: Reflections on process and value. Int J Soc Res Meth..

[64] Survey Monkey Inc. http://www.surveymonkey.com.

[65] Hobbart JC, Cana SJ, Zajicek JP, Thompson AJ (2007). Rating scales as outcome measures for clinical trials in neurology: problems, solutions, and recommendations. Lancet Neurol..

[66] Norusis MJ (2007). SPSS 15.0 Advanced statistical procedures companion.

[67] Tabachnick BG, Fidell LS (2007). Using multivariate statistics.

[68] Twisk JWR (2011). Applied multilevel analysis.

[69] Zorn CJW (2001). Generalized estimating equation models for correlated data: a review with applications. Am J Polit Sci.

[70] Mamode Khan N, Heenaye M (2011). A computationally efficient algorithm to solve generalized method of moments estimating equations based on secant-vector divisions procedure. Intern J Sci Stat Comp.

[71] Lipsitz SR, Fitzmaurice GM, Orav EJ, Laird NM (1994). Performance of generalized estimating equations in practical situations. Biometrics.

[72] Orme D (2010). Getting started with conjoint analysis: strategies for product design and pricing research.

[73] Lemay MA, Sa C (2014). The use of academic research in public health policy and practice. Res Evaluat..

[74] Albert M, Fretheim A, Maiga D (2007). Factors influencing the utilization of research findings by health policy-makers in a developing country: the selection of Mali’s essential medicines. Health Res Policy Syst..

[75] Whitehead M, Petticrew M, Graham H, Macintyre S, Bambra C, Egan M (2004). Evidence for public health policy on inequalities 2: assembling the evidence jigsaw. J Epidemiol Community Health..

[76] Lavis JN, Ross SE, Hurley JE, Hohenadel JM, Stoddart GL, Woodward CA (2002). Examining the role of health services research in public policymaking. Milbank Q.

[77] Lewis S, Bosch-Capblanch X, Oliver S, Aki EA, Vist G, Lavis J (2012). Guidance for evidence-informed policies about health systems: assessing how much confidence to place in the research evidence. PLoS Med.

[78] Daly J, Willis K, Small R, Green J, Welch N, Kealy M (2007). A hierarchy of evidence for assessing qualitative research. J Clin Epidemiol..

[79] Ross S, Lavis J, Rodriguez C, Woodside J, Denis J (2003). Partnership experiences: involving decision-makers in the research process. J Health Serv Res Policy.

