# Supplementary material for: Using conjoint analysis to develop a system to score research engagement actions by health decision makers
Source: Health Res Policy Syst. 2015 Apr 26;13:22. doi: 10.1186/s12961-015-0013-z (PMC4443514; doi:10.1186/s12961-015-0013-z)
Supplement: Additional file 1: — SAGE: Assessing the use of research in policy products - Interviewer’s guide. [file 12961_2015_13_MOESM1_ESM.pdf]

SAGE: Assessing the use of research in policy products - Interviewer's guide

FOR INTERVIEWER:

- *This interview is designed to be carried out as a friendly, open conversation about the development of a particular policy document.*
- *The purpose is to explore how research contributed to the development of the document, understanding that research can be used in ways that complicated, subtle, multilayered. Policy making is an intricate, complex, non-linear process that has many interacting components and competing masters. The aim of the interview, therefore, is to understand and accurately describe research use, rather than to assess research use.*
- *Interviews are to be audio recorded.*
- *Except where explicitly noted in Question 19, the interviewer will not use the scores with interviewees.*
- *The interviewer is not expected to mark each question using the scores. They are included in this guide to provide information that may be useful for phrasing questions and selecting prompts.*
- *The structure below is a guide. The conversation might not always be this linear, however the interviewer should ensure that all questions are addressed before the interview concludes. We expect that interviews will take up to 1 hour.*
- *The prompts in green text are for use with evaluation documents. They can be used as supplementary questions but should not replace the main question they are attached to.*
- *'Skip logic' instructions are shown in blue text*

Some interviewees ask how we are defining research, policy and policy documents. These definitions are also provided in the participant information:

Research is...

Analyses of quantitative or qualitative data, or theory, found in peer reviewed papers, technical monographs or books, or in grey literature such as internal studies and evaluations, and reports on authoritative websites.

Advice from researchers is considered to be *research-informed information*, but not research per se.

We use the terms *research*, *research findings* and *research evidence* interchangeably.

Policy or programs are...

A formal statement or action developed by a government agency or statutory body in response to an identified problem. This includes state-wide or national legislation, policies, programs, directives, protocols, guidelines, and service models.

A policy document is...

A review, report, discussion paper, draft or final policy, formal directive, program plan, strategic plan, ministerial brief, budget bid, service agreement, implementation plan, guideline or protocol with a focus on health service or program design, delivery, evaluation or resourcing.

(1) Date of interview: **DD** **MM** **YYYY**

(2) Organisation

(3) Policy Document Name

(4) Interviewee Name

(5) Interviewee Number (for this document)

1 ◯ 2 ◯

(6) Mode of interview

Face to face ◯ Phone ◯

(7) Audio recorded

Yes ◯ No ◯

The Interview

**"Thank you for agreeing to participate in a SAGE interview. Is this time still convenient for you? As you know, during this interview we will be asking about how research was used in the development of a particular policy or program document."**	*Confirm it is still OK to record.*<br><br>*Advise interviewee that you are turning recorder on.*<br><br>*Note organisation, name of participant and title of policy document on top of file*

Question	Actions and scoring prompts	Comments	Additional prompts
1. **Can you tell me about the document that we are going to discuss today?**	Confirm that document:<br>☐ has been signed off or issued in the last X months; and<br>☐ relates to a policy or program about health services or health services resourcing	Ideally, the interviewee will have provided a copy of the policy document before the interview but this is not always be possible. The policy-makers' description of the document will provide useful context for the rest of the interview.	*What is the purpose of this document? What policy issue/issues does it address?*<br>**For evaluation documents: What was the purpose of the evaluation? How did you evaluate the program?**
**2. Can you explain for me why this document was selected for a SAGE interview?**		Agencies are asked to select 4 documents for SAGE interviews that best demonstrate the agency's capacity to use research. This question is to help understand how that applies to this document.	
**3. What was your role in the development of this document?**		Understanding the role of the interviewee is very important to the validity of the interview. Was their role central, marginal, advisory? Did they do the document development, contribute to it, direct it?	

Question	Actions and scoring prompts	Comments	Additional prompts
**4. Did you or others look for research to inform development of this document?**	☐ No - ***go to question 12.***<br>☐ Yes	***For evaluation documents: Was research sought to inform the methods of the evaluation or to select outcomes of interest, or to identify existing measurement tools?***	*If NO: what other sources of information were used? Was there any reason not to (look for research)? Eg no time, didn't feel the need, already had experience in the area?*
**5. Can you describe for me how you or others looked for research to inform this document?**	• Academic literature or systematic review databases?<br>• Consult with research experts, librarian or reference groups to find research?<br>• Search grey literature and data sources, e.g. government websites?<br>• Look through reference lists, citation databases or reference libraries?<br>• Use research that was already known, on hand or provided by colleagues?<br>• Use generic search engines such as Google or Google Scholar?	We want to know what methods were used to find evidence. Was this process systematic or serendipitous? Was the search broad and rigorous, or narrow and limited?<br>*The scoring notes can be used as an informal checklist or prompts*	*What was actually done to get research? Where did you look?*<br>*What kinds of sources were searched, eg academic databases, government websites, Google, sources they already knew about? Who did the searching?*<br>*Do you think you found everything that might have been helpful?*
**6. Was any research found?**	☐ No - ***go to question 12.***<br>☐ Yes		*If no, can you explain why not? Eg nothing relevant, nothing of any quality.*
**7. If yes, what types of research were found?**	• Primary research studies; such as reports of trials published in research journals<br>• Secondary research articles (reviews) such as systematic reviews or research summaries<br>• Technical monographs or books<br>• Govt. reports or other unpublished (grey) literature, including evaluations of policies or programs from other organisations<br>• Internal data or evaluations<br>• Data from registries/databases such as the Australian Institute of Health and Welfare, etc<br>• Other:	Again, the scoring list can be used as a checklist or talking points to pinpoint what types of research were found, but use this sparingly. If it is apparent that very little searching was done and minimal research was found, do not labour the point.	*Was this research recent?*

Question	Actions and scoring prompts	Comments	Additional prompts
**“We've talked about how you accessed research and what you found, now I would like to talk about if or how you assessed the relevance and quality of that research.”**			
**8. Thinking first about the relevance of the research to this document, how relevant was the research that was found?**	1 – Not relevant enough to be able to be applied in our policy context, population, etc<br>2 – Some relevance and possibly able to be applied in our policy context, population, etc<br>3 – Relevant and likely to be able to be applied in our policy context, population, etc<br>4 – Very relevant and very likely to be able to be applied to our policy context, population, etc<br>5 – Directly applicable to our policy context, population, etc	By relevance we mean how appropriate and applicable the research was for the particular topic the interviewee was trying to address.	*Was the research fit for purpose in that it helped you answer your program or policy question?*<br>*Was it a fit for your population and context?*<br>*Was it sufficiently up-to-date for your needs?*<br>*Was it formatted for action, e.g. did it include policy recommendation, were they feasible?*
**9. How did you, or others, work out whether the research was relevant or not?**	Was an assessment made of whether the research was:<br>• Applicable to the policy context or issue?<br>• Actionable/feasible?<br>• Consistent with previous research?<br>• Compatible with organisational values/knowledge?<br>Did the policymaker:<br>• Consult with experts to assess relevance?<br>• Use criteria or a structure appraisal guide?<br>• Undertake these actions as part of a predefined strategy, or was it ad hoc/intuitive?	The score categories can be used as an informal checklist or prompts if the situation warrants it.<br>Multiple methods may be applicable<br>This will be scored on how transparent and systematic the process was.	*How was the relevance of the research determined? Were particular methods used or was it an intuitive process? Were there any specific criteria? Was there a standard process or was it ad hoc? Who did the assessment?*
**10. Thinking now about the quality or reliability of the research, what was the quality of the research that was found?**	0 – No research<br>1 – Low quality research<br>2 – Moderate quality research<br>3 – High quality research<br>4 – Variable quality research<br>5 - Don't know	By quality or reliability we mean how scientifically robust the research was	*Was the design robust enough to lead to accurate conclusions, e.g. was the sample size sufficient, were the data collection methods appropriate? Were threats to validity addressed?*

Question	Actions and scoring prompts	Comments	Additional prompts
**11. How did you, or others, evaluate the quality of the research?**	• Appraisal using systemic, structured guide<br>• Appraisal assessed the level of evidence<br>• Appraisal based on assessment by expert<br>• Appraisal or research design or conclusions<br>• Appraisal of author/source credibility<br>• Appraisal of citations or references in research/policy/program docs<br>• Transparently documented<br>• Pre specified process or intuitive / ad hoc	This will be scored on how transparent and systematic the process was.<br><br>Again, the score categories can be used as an informal checklist or prompts if the situation warrants it.	*How was the quality of the research determined? Were particular methods used or was it an intuitive process? Were there any specific criteria? Was there a standard process or was it ad hoc? Who did the assessment?*
**12.** *Given the amount and quality of the research that you found…* **Do you, or others, plan to commission, conduct or advocate for more research as part of development of this document?**	• Commission or partner with researchers to conduct research project or analysis of data<br>• Evaluate the program or policy in question<br>• Internally conduct research or analysis of data<br>• Form a working group, consulted with stakeholders or advisory committee<br>• Advocate: lobby, or explain need for research in press release or to researchers<br>• Clear intentions/plans or Uncertain or No intention/plans	Once again, the score categories can be used as an informal checklist or prompts, but only if it is appropriate to the situation (there must be no suggestion that the interviewee SHOULD have done any of these things).<br>**This includes plans to do follow-up research on interesting findings in an evaluation report, for example.**	*If yes, what were they? If no, why not?*<br><br>*[If working groups are mentioned…] Did they include 'experts' e.g. researchers, clinicians, specialist practitioners who would have to stay on top of the literature?*

If the answer to Q4 was NO (i.e. research was not sought at all) go to Q15 now

"Having explored the way that you or others looked for and evaluated research, I would now like to talk about how research was actually used"

**13. Did research inform the development of this document in any way?**	☐ No* - *go to question 15.*<br>☐ Yes	This is the core question of the interview. Spend time here unpacking all the ways in which research contributed to the development of the document.<br>*If the initial answer is no, please explore how it was developed to ensure this is accurate, particularly explore indirect research use as below.	*If not, why not? What did inform the document? If any research was found, was it used in any way at all?*

Question	Actions and scoring prompts	Comments	Additional prompts
"We're interested in the nitty gritty of how research gets into policy and program documents"			
14. **Can you tell me how you (or others) used research in the development of this document?**		Keep this as an open question to start with…	*Can you describe how you used research in that way?*
**I'm going to talk about four different ways that research can be used and after each one I'd like you to tell me if you used research this way or not**		We need to know if research contributed instrumentally, conceptually, tactically, or if it has been imposed.	*Can you describe how you used research in this way?*
• Was research used to help you understand how to think about an issue?	Did research:<br>• inform thinking about the background to a health issue<br>• help you understand the program/policy context (e.g., target population, setting, feasibility)<br>• suggest policy priorities (e.g. by suggesting which issues need action)<br>• suggest alternative strategies<br>• help you design an evaluation	Conceptual use of research is when the research provides indirect 'knowledge for understanding', helps people to grasp issues, influences thinking but without documentable, direct impact<br><br>**This includes using research to inform background thinking about how to design and conduct an evaluation, to select outcomes, or to inform the development of evaluation tools.**	*Can you describe how you used research in that way?*
• Was research used to make decisions about program/ policy content or direction?	Did research:<br>• Directly inform policy/program decisions, (e.g. help you to identify priorities or alternative strategies)<br>• Directly feed into this document	Instrumental use of research provides 'knowledge for action', informing policy decisions and document content directly<br><br>**This includes use of research to determine evaluation methods, to select outcomes, or to identify existing tools to use.**	*Can you describe how you used research in that way?*

Question	Actions and scoring prompts	Comments	Additional prompts
• Was research used to persuade others to a point of view or course of action?	Was research used to:<br>• Provide hard evidence/ammunition to persuade stakeholders to support or act upon an existing decision or view<br>• Support, confirm, back up, or justify an established position, decision, or view<br>• Inform the stakeholders about the key issues relating to the policy issue	Tactical use of research gives credibility to an existing policy/program decision, often by persuading others to support it	*Can you describe how you used research in that way?*<br><br>*[please probe the interviewee as to which stakeholders they were trying to persuade/inform/ justify the decision to – if these were not mentioned voluntarily]*
• was research used because your organisation required you to use research?	Was research used because:<br>• The organisation encourages the use of research<br>• The organisation expects research to be used (i.e., it is regarded as best practice)<br>• The organisation mandates research use (there is usually some checklist or documented process if this is the case)	Imposed research is where there are organisational or regulatory requirements to use it in some way.	*Can you describe how you used research in that way?*
• Was research use in any other ways?			*If so what other ways?*
**15. Were there any barriers to using research in the development of this document?**	☐ No* - ***go to question 16.***<br>☐ Yes<br><br>0 – No barriers<br>1 – Minimal impact on research use<br>2 – Limited impact on research use<br>3 – Moderate impact on research use<br>4 – Substantial impact on research use<br>5 – Extensive impact on research use	*If the initial answer is no, please explore this to ensure this is accurate.<br><br>We are interested in all kinds of barriers, including those at the individual, team, agency, political, topical etc level. Participants are likely to identify "time". If so please explore to determine the cause of the time pressure, e.g. does this relate to low priority on research use activities, or unexpected external factors, or etc.<br><br>Please also explore HOW the barrier impacted on the development process.	*What impact did they have?*<br><br>*How much of a problem were they?*

Question	Actions and scoring prompts	Comments	Additional prompts

If answer to Question 13 was NO (i.e. research did not inform the development of this document in any way) go to Question 17

Question	Actions and scoring prompts	Comments	Additional prompts
**16. Were there any things that helped the use research *(facilitators/enablers)* in the development of this document?**	☐ No* - ***go to question 17.***<br>☐ Yes<br>Facilitators might relate to:<br>• Individual skills/knowledge<br>• Team attributes<br>• Agency level attributes<br>• Political factors<br>• Policy/program topic factors	*If the answer is no, explore to ensure this is accurate. E.g. did they knew how to find research? Was it easy to access?<br>Please also explore HOW the facilitator impacted on the development process.<br>Facilitators may be specific to this document and wouldn't always apply.	*What impact did this have?*<br>*How much help were they?*
**17. Could research have been more helpful in the development of this document?**<br>*If yes, how?*	☐ No - ***go to question 18.***<br>☐ Yes	Note: This may overlap with barriers above, but is important to determine whether there was potential for research to be more useful, or whether that was not possible.	*Were there any gaps/weaknesses/limitations in the research* or the way that it was presented that limited its usefulness to this *document? Was there anything about the nature of the policy process that limited the opportunity for research to contribute?*
**18. Do you have any concerns about how research was or wasn't used in the development of this document?**<br>*If yes, what are they?*	☐ No - ***go to question 19.***<br>☐ Yes	Tactical or imposed research use, in particular, may not lead to improved policy. This question aims to identify whether there were any negative consequences of the use (or non-use) of research. This might include negative reactions to imposed research use. Poorly understood/communicated/appraised or generalised research might result in overconfidence in the benefit of particular initiatives or less than ideal policy.	

Question	Actions and scoring prompts	Comments	Additional prompts
**19. On a scale of zero to five; with zero being "Played no role" and five being "Essential", how would you rate the importance of research in the development of this document?**	0 – Not at all important – played no role<br>1 – Minimal importance – very little influence on document<br>2 – Limited importance – little influence on document<br>3 – Moderate – some influence on document<br>4 – Important – substantial influence on document<br>5 – Essential – extensive influence on document	WHEN PARTICIPANT PROVIDES RESPONSE, CHECK THAT THE DESCRIPTION OF THE NUMERICAL RATING MATCHES WITH THEIR INTERPRETATION OF THAT RATING	What's your assessment of the importance of the contribution of research to the development of the policy or program **(or in the design of the evaluation)** overall?
**20. How representative is this document of policy /program development in your organisation (particularly in relation to the use of research)?**		Agencies are asked to select 4 documents for SAGE interviews that best demonstrate the agencies capacity to use research. This question is to help understand whether this document is an exceptional case, or is in line with usual practice in the agency.	
**21. Do you think the results of this evaluation will impact on future program or policy development?**		**This question should be asked only if the document is an evaluation**	If yes, how? If not, why not?
**22. Do you have any other comments about the use of research in the development of this document?**			
**Thank the participant for their time and the information/insights they have provided.**	• Note length of interview<br>• Copy the audio file to: *insert location*<br>• Use naming convention: *insert naming convention*<br>• Note interview completion in *management database.*		
