# Supplementary material for: Using conjoint analysis to develop a system to score research engagement actions by health decision makers
Source: Health Res Policy Syst. 2015 Apr 26;13:22. doi: 10.1186/s12961-015-0013-z (PMC4443514; doi:10.1186/s12961-015-0013-z)
Supplement: Additional file 2: — Profiles and questions in the SAGE ONLINE survey. [file 12961_2015_13_MOESM2_ESM.pdf]

Profiles and questions in the SAGE ONLINE survey

1. Searching for research

Listed below are eight different scenarios reflecting how a policymaker went about locating research to inform the development of a policy document.
Research includes both academic and grey literature resources.
Please rate, on the 1-9 scale, the **extent to which each scenario represents a *limited, moderate, or extensive*** strategy for searching for research.

1	2	3	4	5	6	7	8	9
Limited			Moderate			Extensive		

Profile	Rating out of 9
1. The policymaker...<br>• Searched grey literature sources (e.g., OpenGREY, ProQuest International, WHO)<br>• Consulted experts and/or librarians to identify relevant research<br>• Used search engines (e.g., Google)<br>• Examined reference lists of articles or EndNote database	
2. The policymaker…<br>• Searched academic literature databases (e.g., Medline, Cochrane Library) or libraries<br>• Used research that was already on hand<br>• Used search engines (e.g., Google)<br>• Examined reference lists of articles or EndNote database	
3. The policymaker...<br>• Used research that was already on hand or given by others<br>• Consulted experts and/or librarians to identify relevant research<br>• Examined reference lists of articles or EndNote database	
4. The policymaker...<br>• Searched academic literature databases (e.g., Medline, Cochrane Library) or libraries<br>• Searched grey literature sources (e.g., OpenGREY, ProQuest International, WHO)<br>• Examined reference lists of articles or EndNote database	
5. The policymaker...<br>• Searched academic literature databases (e.g., Medline, Cochrane Library) or libraries<br>• Consulted experts and/or librarians to identify relevant research<br>• Used search engines (e.g., Google)	
6. The policymaker...<br>• Searched grey literature sources (e.g., OpenGREY, ProQuest International, WHO)<br>• Used research that was already on hand<br>• Used search engines (e.g., Google)	
7. The policymaker...<br>• Searched academic literature databases (e.g., Medline, Cochrane Library)	

or libraries<br>• Searched grey literature sources (e.g., OpenGREY, ProQuest International, WHO)<br>• Used research that was already on hand<br>• Consulted experts and/or librarians to identify relevant research	
8. The policymaker...<br>• Did not undertake any of the aforementioned search strategies	

2. Research obtained and used

Listed below are eight different scenarios reflecting the types of research a policymaker may obtain when developing a policy document.

Please rate, on the 1-9 scale below, the **extent to which each scenario represents *a limited, moderate, or extensive* accessing of available research**.

1	2	3	4	5	6	7	8	9
Limited			Moderate			Extensive		

Profile	Rating out of 9
1. The policymaker found...<br>• Systematic reviews and/or meta-analyses<br>• Primary research and/or theoretical articles<br>• Books and/or technical monographs<br>• Unpublished research and/or conference resources<br>• Internal policies, evaluations, or data<br>• Policies, evaluations, or data from external organisations or registries<br>• Recent research from the above categories	
2. The policymaker accessed...<br>• Primary research and/or theoretical articles<br>• Policies, evaluations, or data from external organisations or registries<br>• Recent research from the above categories	
3. The policymaker accessed...<br>• Books and/or technical monographs<br>• Internal policies, evaluations, or data<br>• Recent research from the above categories	
4. The policymaker accessed...<br>• Systematic reviews and/or meta-analyses<br>• Unpublished research and/or conference resources<br>• Recent research from the above categories	
5. The policymaker accessed ...<br>• Unpublished research and/or conference resources<br>• Internal policies, evaluations, or data<br>• Policies, evaluations, or data from external organisations or registries<br>• Dated research from the above categories	
6. The policymaker accessed ...<br>• Systematic reviews and/or meta-analyses<br>• Books and/or technical monographs<br>• Policies, evaluations, or data from external organisations or registries<br>• Dated research from the above categories	
7. The policymaker accessed ...<br>• Systematic reviews and/or meta-analyses<br>• Primary research and/or theoretical articles<br>• Internal policies, evaluations, or data<br>• Dated research from the above categories	
8. The policymaker accessed ...	

• Primary research and/or theoretical articles<br>• Books and/or technical monographs<br>• Unpublished research and/or conference resources<br>• Dated research from the above categories	

3. Appraising the relevance of research

Listed below are eight different scenarios reflecting how a policy maker went about assessing the applicability of research to the current policy question

Please rate, on the 1-9 scale below, the **extent to which each scenario represents a *limited, moderate, or extensive* appraisal of research relevance**.

1	2	3	4	5	6	7	8	9
Limited			Moderate			Extensive		

Profile	Rating out of 9
1. The policymaker...<br>• Assessed whether research recommendations were actionable and/or feasible<br>• Assessed whether research was compatible with his/her OR the organisation's values, knowledge, or experience<br>• Consulted experts to assess relevance<br>• Undertook these actions as part of a pre-specified strategy	
2. The policymaker...<br>• Assessed whether the research was applicable to the policy context or policy issue<br>• Assessed whether the research was consistent with previous research on the issue<br>• Consulted experts to assess relevance<br>• Undertook these actions as part of a pre-specified strategy	
3. The policymaker...<br>• Assessed whether the research was consistent with previous research on the issue<br>• Assessed whether research was compatible with his/her OR the organisation's values, knowledge, or experience<br>• Undertook these actions as part of a pre-specified strategy	
4. The policymaker...<br>• Assessed whether the research was applicable to the policy context or policy issue<br>• Assessed whether research recommendations were actionable and/or feasible<br>• Undertook these actions as part of a pre-specified strategy	
5. The policymaker...<br>• Assessed whether the research was applicable to the policy context or policy issue<br>• Assessed whether research was compatible with his/her OR the organisation's values, knowledge, or experience<br>• Consulted experts to assess relevance<br>• Undertook these actions as part of an ad-hoc or intuitive strategy	
6. The policymaker...<br>• Assessed whether research recommendations were actionable and/or feasible	

• Assessed whether the research was consistent with previous research on the issue<br>• Consulted experts to assess relevance<br>• Undertook these actions as part of an ad-hoc or intuitive strategy	
7. The policymaker...<br>• Assessed whether the research was applicable to the policy context or policy issue<br>• Assessed whether research recommendations were actionable and/or feasible<br>• Assessed whether the research was consistent with previous research on the issue<br>• Assessed whether research was compatible with his/her OR the organisation's values, knowledge, or experience<br>• Undertook these actions as part of an ad-hoc or intuitive strategy	
8. The policymaker...<br>• Did not undertake any of the above-mentioned actions to assess relevance<br>• Assessment of relevance was part of an ad-hoc or intuitive strategy	

4. Appraising the quality of research

Listed below are eight different scenarios reflecting how a policy maker went about assessing the credibility, validity, or rigour of research and its conclusions

Please rate, on the 1-9 scale below, the **extent to which each scenario represents a *limited, moderate, or extensive* appraisal of research quality**.

1	2	3	4	5	6	7	8	9
Limited			Moderate			Extensive		

Profile	Rating out of 9
1. The policymaker...<br>• Assessed whether the research design or conclusions were valid<br>• Evaluated whether the research design or conclusions were described clearly and comprehensively<br>• Assessed the credibility of the source of the research<br>• Checked whether the research cited, or was referenced in other high-quality research or policy documents<br>• Consulted experts to assess quality<br>• Assessed the level of evidence of the research<br>• Undertook these strategies as part of a pre-specified strategy	
2. The policymaker...<br>• Assessed the credibility of the source of the research<br>• Assessed the level of evidence of the research<br>• Undertook these strategies as part of a pre-specified strategy	
3. The policymaker...<br>• Evaluated whether the research design or conclusions were described clearly and comprehensively<br>• Consulted experts to assess quality<br>• Undertook these strategies as part of a pre-specified strategy	
4. The policymaker...<br>• Assessed whether the research design or conclusions were valid<br>• Checked whether the research cited, or was referenced in other high-quality research or policy documents<br>• Undertook these strategies as part of a pre-specified strategy	
5. The policymaker...<br>• Checked whether the research cited, or was referenced in other high-quality research or policy documents<br>• Consulted experts to assess quality<br>• Assessed the level of evidence of the research<br>• Undertook these actions as part of an ad-hoc or intuitive approach	
6. The policymaker...<br>• Assessed whether the research design or conclusions were valid<br>• Evaluated whether the research design or conclusions were described clearly and comprehensively<br>• Assessed the level of evidence of the research<br>• Undertook these actions as part of an ad-hoc or intuitive approach	

7. The policymaker...<br>• Assessed whether the research design or conclusions were valid<br>• Assessed the credibility of the source of the research<br>• Consulted experts to assess quality<br>• Undertook these actions as part of an ad-hoc or intuitive approach	
8. The policymaker...<br>• Evaluated whether the research design or conclusions were described clearly and comprehensively<br>• Assessed the credibility of the source of the research<br>• Checked whether the research cited, or was referenced in other high-quality research or policy documents<br>• Undertook these actions as part of an ad-hoc or intuitive approach	

5. Generating new research and/or analyses

Listed below are eight different scenarios reflecting a policymaker's plans or intentions to commission or undertake new research, evaluations, or analyses to inform the current and/or future policy.

Please rate, on the 1-9 scale below, the **extent to which each scenario represents a *limited, moderate, or extensive* strategy for generating new research**.

1	2	3	4	5	6	7	8	9
Limited			Moderate			Extensive		

Profile	Rating out of 9
1. The policymaker...<br>• Mentioned thorough research generation activities<br>• Mentioned less intensive research activities (e.g., advisory groups, formalised meetings).<br>• Had uncertain intentions to generate or commission this new research<br>• Advocated for future research to be undertaken	
2. The policymaker...<br>• Mentioned less intensive research activities (e.g., advisory groups, formalised meetings).<br>• However, had no intentions to generate or commission this new research<br>• Advocated for future research to be undertaken	
3. The policymaker...<br>• Mentioned thorough research generation activities<br>• However, had no intentions to generate or commission this new research<br>• Advocated for future research to be undertaken	
4. The policymaker...<br>• Had explicit intentions to generate or commission new research<br>• However, did not mention any specific research generation activities<br>• Advocated for future research to be undertaken	
5. The policymaker...<br>• Mentioned thorough research generation activities<br>• Mentioned less intensive research activities (e.g., advisory groups, formalised meetings).<br>• Had explicit intentions to generate or commission this new research	
6. The policymaker...<br>• Mentioned less intensive research activities (e.g., advisory groups, formalised meetings).<br>• However, had no intentions to generate or commission this new research	
7. The policymaker...<br>• Mentioned thorough research generation activities<br>• However, had no intentions to generate or commission this new research<br>• Consulted experts and/or librarians to identify relevant research	
8. The policymaker...<br>• Did not mention any specific research generation activities<br>• Had uncertain intentions to generate or commission new research	

6. Interacting with researchers

Listed below are eight different scenarios reflecting how a policy maker went about interacting with researchers to inform the development of the current policy

Please rate, on the 1-9 scale below, the **extent to which each scenario represents a *limited, moderate, or extensive* strategy for interacting with researchers**.

1	2	3	4	5	6	7	8	9
Limited			Moderate			Extensive		

Profile	Rating out of 9
1. The policymaker...<br>• Engaged in thorough collaborative activities with researchers<br>• Engaged in sporadic contact with other researchers<br>• Actively initiated these interaction activities	
2. The policymaker...<br>• Engaged in sporadic contact with researchers<br>• Actively initiated these interaction activities	
3. The policymaker...<br>• Engaged in thorough collaborative activities with researchers<br>• Engaged in less intensive interactions with other researchers<br>• Actively initiated these interaction activities	
4. The policymaker...<br>• Engaged in less intensive interactions with researchers<br>• Actively initiated these interaction activities	
5. The policymaker...<br>• Engaged in thorough collaborative activities with researchers<br>• Engaged in less intensive interactions with other researchers<br>• Engaged in sporadic contact with other researchers<br>• Did NOT pre-plan or actively initiate these interaction efforts	
6. The policymaker...<br>• Engaged in less intensive interactions with other researchers<br>• Engaged in sporadic contact with other researchers<br>• Did NOT pre-plan or actively initiate these interaction efforts	
7. The policymaker...<br>• Engaged in thorough collaborative activities with researchers<br>• Did NOT pre-plan or actively initiate these interaction efforts	
8. The policymaker...<br>• Did NOT mention any specific interaction activities with researchers<br>• Did NOT pre-plan or actively initiate any particular interactions	
