# Supplementary material for: Using conjoint analysis to develop a system to score research engagement actions by health decision makers
Source: Health Res Policy Syst. 2015 Apr 26;13:22. doi: 10.1186/s12961-015-0013-z (PMC4443514; doi:10.1186/s12961-015-0013-z)
Supplement: Additional file 3: — SAGE: Scoring tool. [file 12961_2015_13_MOESM3_ESM.pdf]

CIPHER

saxinstitute

SAGE scoring tool

© 2015 The Sax Institute

Instructions

1. This scoring guide requires you to rate 6 types of research engagement actions
2. Each construct is listed on a separate page. Each page is equivalent to a checklist
   - Each item on the checklist refers to a behaviour or action
3. You will use the checklist to score policymakers' responses to an interview (i.e., the SAGE interview) about a specific policy document. You will also have the policy document itself to help you score.
   - The interview is divided into 6 sections, corresponding to 6 research engagement actions in this scoring guide
4. How to use this scoring guide with the interview
   - Read the policymakers' responses to a particular section (e.g., "*searching for research*")
   - Go to the page in this scoring guide corresponding to that section (e.g., "*I: searching for research*", p.3)
   - If the policy maker has performed a particular action, based on their interview response in this section, tick it off on the checklist
   - Please use the **definitions of terms** following each section to help you determine whether the policymaker has performed this action or not. The glossary contains detailed definitions for all terms highlighted in bold within the scoring guide.
5. Add up the scores displayed for each ticked action to obtain a total score for that particular construct

© 2015 The Sax Institute

I: Searching for Research

Did the policymaker:

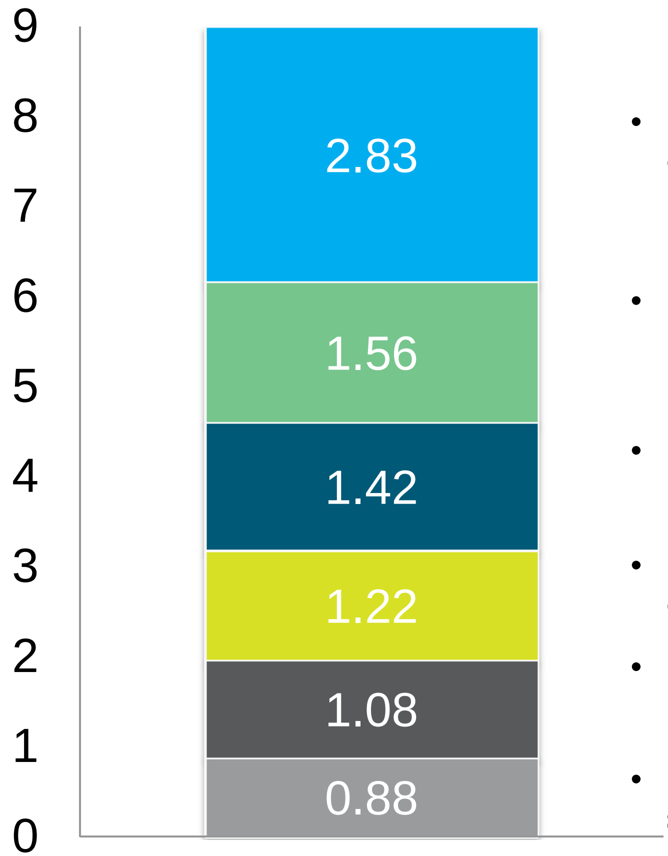

- Search **academic literature databases** or **systematic review databases?** ☐
- Consult **research experts, librarians,** or **reference groups** to help search or identify research? ☐
- Search **grey literature sources?** ☐
- Look through **reference lists** of research document, **citation databases**, or **reference manager databases?** ☐
- Use research that was **on-hand**, in one's **awareness** or **provided by colleagues?** ☐
- Use generic **search engines** or **social media document sharing sites?** ☐

Total

© 2015 The Sax Institute

I: Relevant definitions

SEARCHING FOR RESEARCH: The method used by the policymaker to search for research to inform the development of the policy

ACADEMIC LITERATURE DATABASES: For example: CINAHL, PubMed, SCOPUS, Proquest 5000, EMBASE, LILACS, Science Citations, AMED, Cochrane, MEDLINE, Informit e-Library, Global Health, F1000 Prime, Best Practice, PSYCINFO, CRD, TRIP, Eppi-Centre, EBM Reviews
Also includes databases for systematic reviews (e.g., Cochrane Library)

ACADEMIC LITERATURE: Analyses of quantitative or qualitative data, or theory, found in technical monographs, books, or peer-reviewed resources (i.e., systematic reviews, meta-analyses, rapid reviews, RCTs, non-randomised controlled trials, case studies, case-series, quasi-experiments, non-experimental studies, observational studies, qualitative research studies)

CHANCE FINDINGS/ON-HAND RESEARCH: Research that was on hand and already available or given by colleagues, or research that the policy maker was aware of. Also includes research that was identified through word of mouth, email alerts, or suggested by colleagues within the organisation (stakeholders, executives) or other non-experts

ENDNOTE DATABASE: Organisations may possess an internal EndNote database containing a list of references used in other (related) documents produced by the organisation

© 2015 The Sax Institute

I: Relevant definitions cont'd

EXPERTS: Health experts, researchers, or individuals with experience and knowledge in practice, policy, and research. This includes practitioners, clinicians, service providers, clinical directors and other experts in the health field, as well as health researchers and investigators (individuals, usually scientists, who conduct research in health or other fields)

GREY LITERATURE: Internal studies and evaluations, statistics, qualitative/quantitative data, unpublished studies, dissertations, theses, conference proceedings, conference abstracts, community surveys, and reports

GREY LITERATURE SOURCES: Examples of Grey literature sources include:

- The Agency's local intranet or internal database
- Databases for unpublished studies or dissertations such as ProQuest International, Dissertation Abstracts International, Index to Theses, Australian Digital Theses Program, Canadian Theses and Dissertations, DATAD
- Databases for conference proceedings and abstracts such as ERI, ISTP, INSIDE
- Authoritative websites for government agencies, research organisations, international organisations [e.g., WHO], professional bodies [e.g., Royal Colleges], foundations, archives and clearinghouses
- Grey literature databases such as Public Affairs Information Service, Open-SIGLE database, or OpenGREY
- Databases for unpublished trials in health care such as Cochrane Collaboration's CENTRAL database, ClinicalTrials.gov, or Current Controlled Trials.

LIBRARIAN: A librarian is a person who works professionally in a library, and may hold a degree in librarianship. Librarians are skilled at using library databases and systems to identify relevant research efficiently and thoroughly.

© 2015 The Sax Institute

I: Relevant definitions cont'd

META-ANALYSIS: In statistics, a meta-analysis refers to methods that focus on contrasting and combining results from different studies, in the hope of identifying patterns among study results, sources of disagreement among those results, or other interesting relationships that may come to light in the context of multiple studies. Meta-analyses often (although not always), form key components of systematic reviews

REFERENCE LISTS AND CITATIONS: For example, examining reference lists of high-quality systematic reviews (and other appropriate studies) to identify relevant published, unpublished, or *in-press* research or conducting a citation search using databases such as Web of Science, to identify articles that have cited a particular relevant piece of research

SEARCH ENGINES OR GENERIC DATABASES: e.g., Google Scholar, Yahoo, Bing, Google

SYSTEMATIC REVIEW: A literature review focused on a research question that tries to identify, appraise, select and synthesize all high quality research evidence, to provide an exhaustive summary of the current literature relevant to that question.

© 2015 The Sax Institute

II: Research Obtained and Used

Did the policymaker retrieve and use:

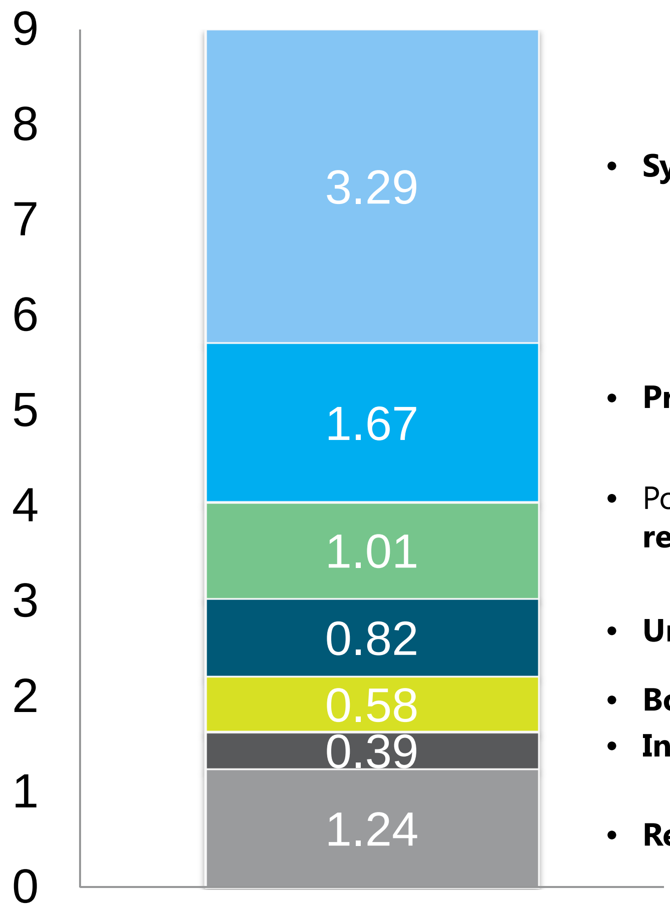

- **Systematic reviews** and/or **meta-analyses?‡**
- **Primary research** and/or **theoretical articles?**
- Policies, evaluations, or data from **external organisations** or **registries?**
- **Unpublished research studies** or **conference resources?**
- **Books** and/or **technical monographs?**
- **Internal** policies, evaluations, or data?
- **Recent (up-to-date)** research from the above categories?

Total

© 2015 The Sax Institute

II: Research Obtained definitions

TYPES OF RESEARCH OBTAINED AND USED: The types of research that were found and used by the policymaker to inform the development of the policy

CONFERENCE RESOURCES: Includes things like conference proceedings, conference abstracts, conference presentations (e.g., PowerPoint slides)

DATED RESEARCH: Research published more than five (5) years ago

INTERNAL POLICIES OR EVALUATIONS: Policies, evaluations, statistics or other data produced by the policy maker's organisations or agency

META-ANALYSIS: In statistics, a meta-analysis refers to methods that focus on contrasting and combining results from different studies, in the hope of identifying patterns among study results, sources of disagreement among those results, or other interesting relationships that may come to light in the context of multiple studies. Meta-analyses often (although not always), form key components of systematic reviews

POLICIES, EVALUATIONS, DATA FROM EXTERNAL ORGANISATIONS: Policies, guidelines, programs, data or evaluations from external organisations, policy agencies in other states or countries

© 2015 The Sax Institute

II: Research Obtained definitions cont'd

PRIMARY RESEARCH: Research published in peer-reviewed journals. For example: Primary research studies in the form of Randomised Controlled Trials (RCTs); pseudo-randomised controlled trials (alternate locations or some other method); comparative studies with concurrent controls and non-randomised allocation, non-equivalent groups designs; cohort studies and other quasi-experiments; case-control studies; interrupted time-series (with or without control groups); comparative studies with historical controls; single-arm studies; case series or case-studies (pre-test or post-test). Also includes descriptive studies examining associations between variables (correlational/regression research, prospective research), observational research, frequencies and patterns (e.g., chi-square; loglinear analysis), and qualitative research.

THEORETICAL RESEARCH: Theoretical articles, commentaries, professional opinions, conceptual papers, or writings describing frameworks or models, published in peer-reviewed journals.

RECENT: Research published in the last five (5) years

REGISTRIES: Data from registries or databases such as the Australian Institute of Health and Welfare, or the Australian Bureau of Statistics

© 2015 The Sax Institute

II: Research Obtained definitions cont'd

SYSTEMATIC REVIEW: A literature review focused on a research question that tries to identify, appraise, select and synthesize all high quality research evidence, to provide an exhaustive summary of the current literature relevant to that question.

TECHNICAL MONOGRAPHS: A specialist work of writing on a single subject or an aspect of a subject, usually by a single author. The purpose is to present primary research and original scholarship on the subject in question.

UNPUBLISHED RESEARCH: Internal or external unpublished data including research reports, research reports from authoritative websites (e.g., the World Health Organisation, dissertations, theses, data for unpublished trials in health care (e.g., clinicaltrials.gov), internally conducted studies and evaluations, community surveys and reports etc.

© 2015 The Sax Institute

III: Appraising Research Relevance

Did the policymaker:

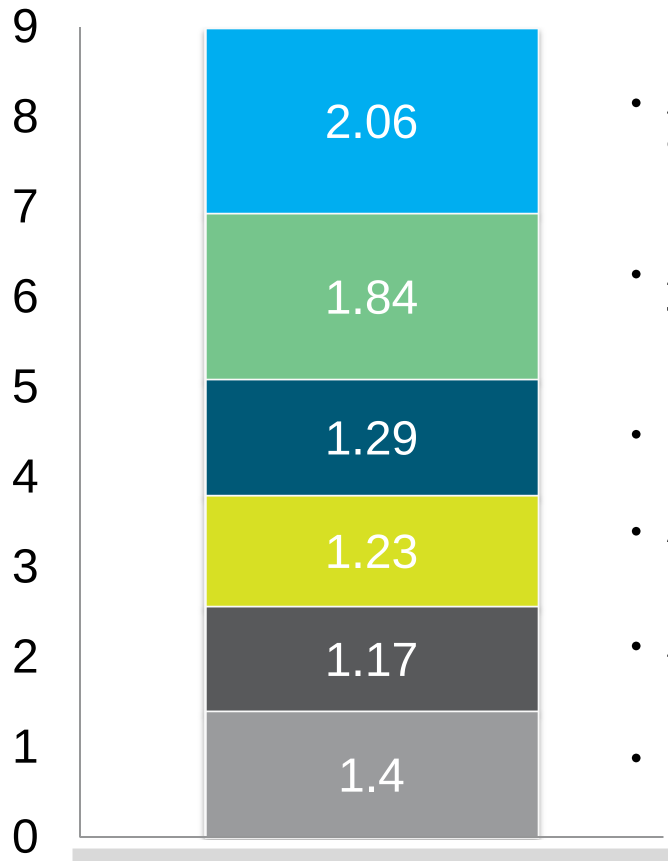

- Assess whether the research was **applicable to the policy context** or **policy issue?**
- Assess whether research recommendations were **actionable** and/or **feasible?**
- Consult **experts** to assess the relevance of research?
- Assess whether the research was **consistent with previous research** on the issue?
- Assess if research was **compatible** with his/her OR the organisation's **values, knowledge, or experience?**
- Undertake these actions as part of a **pre-specified strategy** (as opposed to an **ad-hoc**, **intuitive** strategy)

If the policymaker used a systematic and **structured relevance appraisal guide,** this assumes they undertook items 1, 2, and 6. Thus, give a score of 2.06 + 1.84 + 1.4 = 5.06

Total

© 2015 The Sax Institute

III: Appraising Relevance definitions

APPRAISAL OF RELEVANCE

Assessing whether recommendations, options, or interventions described in a piece of research, is applicable, compatible, or pertinent to the current policy issue

ACTIONABLE: The research clearly and comprehensively conveys a course of action that is direct, practical, efficacious (maximises benefits), safe (minimising harms), flexible, and/or actionable

AD-HOC OR INTUITIVE: Assessment of relevance or quality using the stated actions was intuitive, based on experience, informal, unplanned and/or not transparently documented (i.e., written down, recorded)

FEASIBLE: Sufficient resources (e.g., funds, workers, providers) are available to implement research recommendations. Research recommendations are cost-effective; research recommendations can be easily integrated into current organisational processes and systems OR existing healthcare/service/financing systems without difficulty

POLICY CONTEXT: Features of the policy context include: the current time period, the target setting, the target population, interests of key stakeholders/actors, and the political context

© 2015 The Sax Institute

III: Appraising Relevance definitions cont'd

POLICY ISSUE: The current issue or matter that is the focus of the policy

PRE-SPECIFIED STRATEGY: Assessment of relevance or quality using the stated actions was planned, deliberate, pre-specified, transparently documented (i.e., written down, recorded), and/or purposeful

STRUCTURED RELEVANCE APPRAISAL GUIDELINE: A systematic/structured approach to assessing the applicability of research to the current policy question(s), based on published guidelines (e.g., SUPPORT tool for addressing the applicability of research evidence, Oxman et al., 2009; applicability-transferability guidelines, Wang et al., 2005; replicable interventions, TIDieR, Hoffmann & Glasziou, 2013; Context conceptual frameworks, Dobrow et al., 2004; Brownson et al., 2009) or some other guideline or template produced by the policy maker's organisation.

VALUES, KNOWLEDGE, OR EXPERIENCE: The organisation's values, norms, positions, policies and current projects; The policymaker's current opinions, expectations, knowledge (including that acquired from past education and research), or experience in the area

© 2015 The Sax Institute

IV: Appraising Research Quality

Did the policymaker:

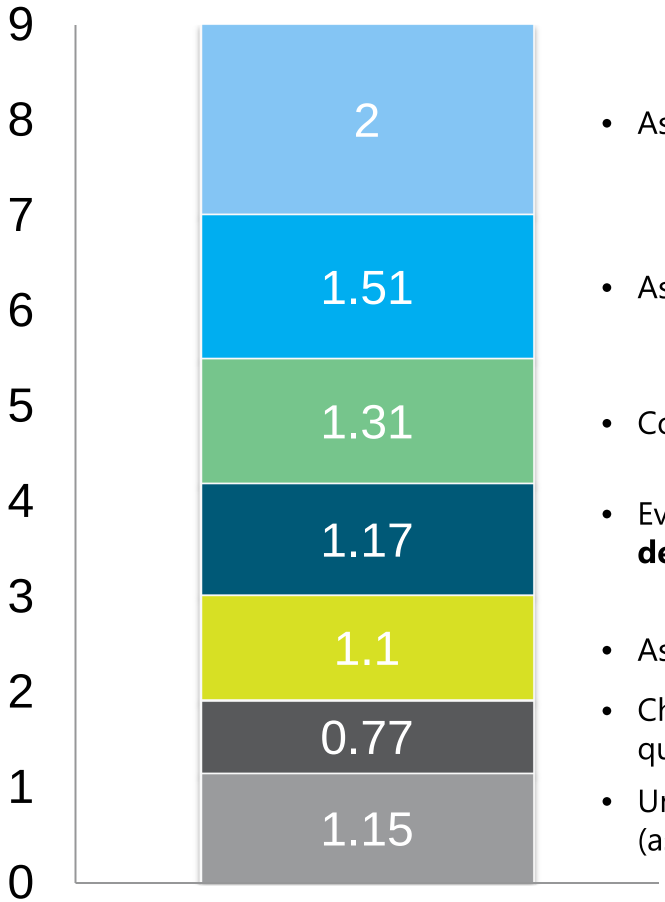

- Assess whether the **design** or **conclusions** of the research were **valid**?
- Assessed the **level of evidence** of the research?
- Consult **experts** to assess quality?
- Evaluate whether the **design** or **conclusions** of the research were **described clearly** and **comprehensively**?
- Assess whether the **source of the research was credible?**
- Check if the research **cited**, or **was referenced in** other high-quality research or policy documents?
- Undertake these actions as part of a **pre-specified strategy** (as opposed to an **ad-hoc**, **intuitive** strategy)

If the policymaker used a **systematic and structured quality appraisal guide,** this assumes they undertook items 1, 2, 4, and 7. Thus, give a score of 2 + 1.51 + 1.17 + 1.15 = 5.83

Total

© 2015 The Sax Institute

IV: Appraising Quality definitions

APPRAISAL OF QUALITY

Assessing the scientific quality, validity, or standard of the research. Quality appraisal refers to evaluating the degree of confidence that can be placed on the conclusions drawn from studies and estimates of effects, and this is based on a number of key factors including its methodology, rigour, validity (i.e., statistical, construct, and internal), and credibility.

CLEAR AND COMPREHENSIVE DESIGN/CONCLUSIONS: The research described the aim, theoretical approach, overall methodology, conclusions, and/or contextual aspects clearly and in detail

LEVEL OF EVIDENCE OF THE RESEARCH: As described by the National Health and Medical Research Council (NHMRC, 1999), levels of evidence is a ranking system that describes the strength of the results measured in a study, based on the design of the study. Levels of evidence, in order of strength (highest to lowest), include:

Ia - Evidence from Meta-analysis/systematic review of all Randomized Controlled Trials
Ib - Evidence from at least one Randomized Controlled Trial
IIa - Evidence from at least one well designed controlled trial which is not randomized
IIb - Evidence from at least one well designed experimental trial
III - Evidence from case, correlation, and comparative studies.
IV - Evidence from a panel of experts

© 2015 The Sax Institute

IV: Appraising Quality definitions cont'd

SOURCE OF THE RESEARCH: Credibility or reputation of the research producer, supplier, journal, or source

STRUCTURED QUALITY APPRAISAL GUIDELINE: A recognised tool for assessing the quality of *quantitative research* (e.g., Threats to validity approach/Study DIAD; GRADE guidelines for assessing the quality of evidence; Cochrane risk of bias criteria; Jadad scale; CASP critical appraisal tool, Public Health Research unit, 2006; AMSTAR or SUPPORT tools for assessing quality of systematic reviews)
AND/OR
A recognised tool to assess the quality of qualitative research (e.g., Mays and Pope, 1995; Boulton et al., 1996; Seale & Silverman, 1997; Quality in Quality Evaluation Framework, National Centre for Social Research, 2003; Daly et al., 2007)

VALID DESIGN AND CONCLUSIONS: Design: Aspects of the study design leading to accurate conclusions and producing the best approximation to the truth. Evaluating appropriateness of the study design (e.g., sample size and recruitment, randomisation, representative samples, sensitive and appropriate data collection methods, appropriate blinding, use of reliable and valid measures, reliable and valid treatment implementation, coherent data analyses) and/or other threats to the validity of conclusions (selection bias, historical factors, selective attrition and mortality, diffusion of treatments, mono-method biases, mono-operation biases, confounding, evaluation apprehension, hypothesis guessing etc.)
Conclusions: Propositions that are accurate, reflect the true state of the world, and produce the best available approximation to the truth.

© 2015 The Sax Institute

V: Generating New Research

Did the policymaker:

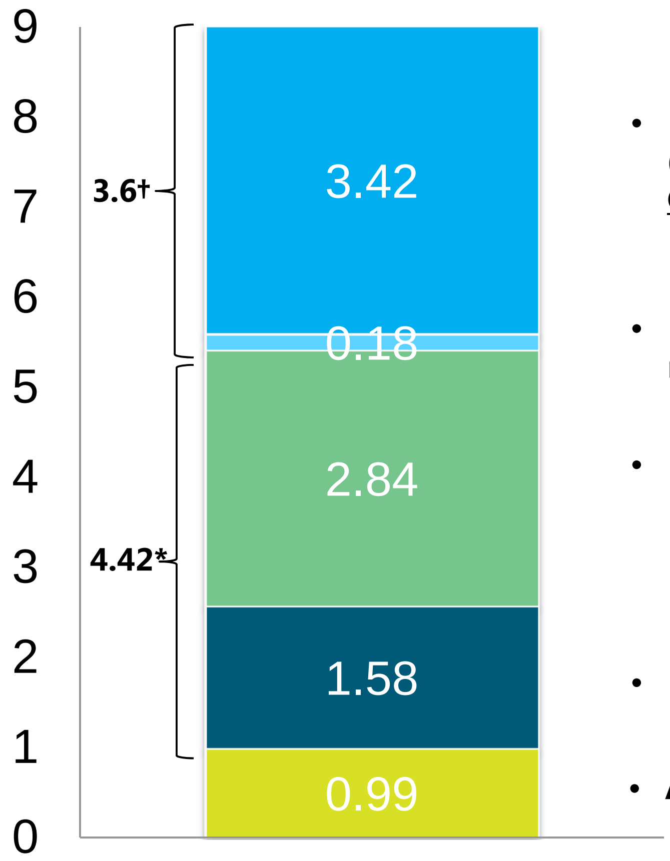

- Express **explicit intentions** to generate or commission new research (to follow-up the current policy)?
  OR state that he/she had **already undertaken** new research?†

OR

- Have **uncertain intentions** to generate or commission new research mentioned?
- Mention **thorough research generation activities?***
- Mention **less intensive research activities**
- **Advocate** for future research to be undertaken?

†If the policymaker has explicit intentions, score 3.42+0.18=3.6
*If he/she has engaged in thorough research activities, 2.84+1.58=4.42

Total

© 2015 The Sax Institute

V: Generating New Research

GENERATE NEW RESEARCH

Commissioning, collaborating in or undertaking new research or new analyses to inform policy/programs

ADVOCATING FOR RESEARCH: Attempting to influence key stakeholders/decision makers/funders to approve the undertaking of research relevant to the policy – this may occur through media campaigns, commissioning research, public speaking, lobbying etc.
EXPLICIT INTENTIONS: If research was yet to be generated, there were explicit or clear intentions/plans to generate/fund this research in the future OR explicitly stated that new research or analyses have already been generated/funded with regard to the policy product.

LESS INTENSIVE RESEARCH ACTIVITIES: Includes activities such as working groups, advisory groups, formalised meetings, and/or workshops with various stakeholders (e.g., executives, users, clinicians, service providers, community groups, other policymakers) to gain information and feedback regarding the policy document. The goal is to gain information from key stakeholders. The findings of these research actiities are not formally analysed or documented. This category does NOT include casual one-to-one meetings with stakeholders.

THOROUGH RESEARCH GENERATION ACTIVITIES: Formalised research activities which includes:
a) Partnered with researchers to conduct a research project and/or analysis of data
b) Undertaking an evaluation of the program or policy in question
c) Internally conducted research project or analysis of data (e.g., focus groups, opinion polls, community surveys) where findings are recorded, analysed, and documented

UNCERTAIN INTENTIONS: If relevant research was yet to be generated, there were indefinite or uncertain intentions/plans to generate or fund this research in the near future

© 2015 The Sax Institute

VI: Interactions with Researchers

Did the policymaker:

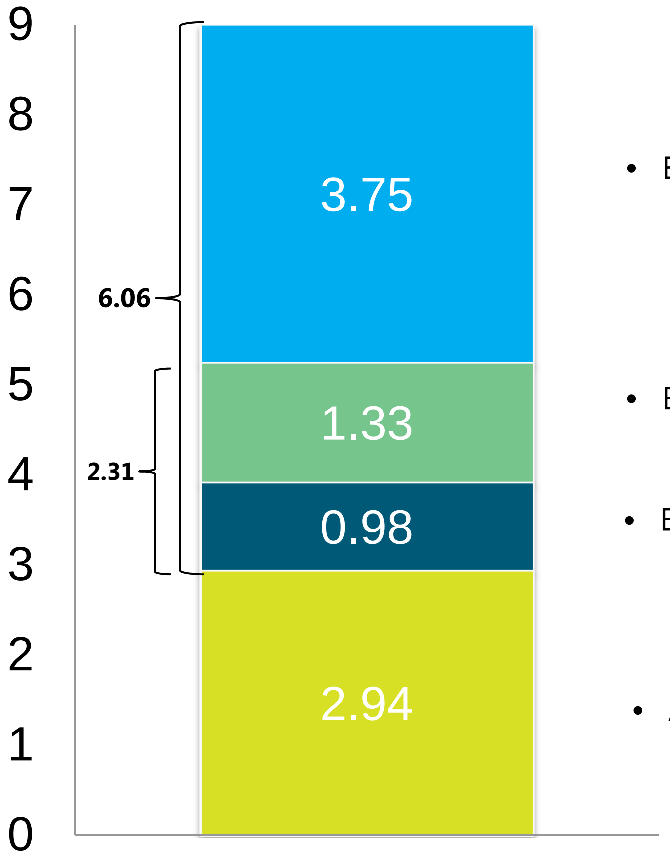

- Engage in **thorough collaborative activities** with researchers?†
- Engage in **less intensive interactions** with (other) researchers?*
- Engage in **sporadic contact** with (other) researchers?
- **Actively initiate** these interaction activities?

†If the policymaker has engaged in thorough collaborative activities, score 3.75+1.33+0.98=6.06
*If the policymaker has engaged in less intensive interactions, score 1.33+0.98=2.31

Total

© 2015 The Sax Institute

VI: Interactions with Researchers definitions

INTERACT WITH RESEARCHERS

Interaction, collaboration and communication with researchers through events, projects, networks, committees, etc.

DELIBERATE/ACTIVELY INITIATED INTERACTIONS: Interaction efforts were deliberate and/or actively initiated by the policy maker

LESS INTENSIVE INTERACTIONS:

a) Contacted researchers to identify other key researchers

b) Posted requests for research in Listservs, mailing lists, or forums targeting relevant researchers

c) Formally supported a research project relevant to the policy (e.g., providing information, access to data, resources)

SPORADIC CONTACTS WITH RESEARCHERS: One-off, inconsistent forms of interaction (e.g., sent an email, made a phone call, liaised with a researcher colleague)

THOROUGH COLLABORATIVE ACTIVITIES

Includes:
a) Collaborating with researchers to identify research priorities and ideas
b) Collaborating with researchers to formulate, conceptualise, and design a research project
c) Commissioned researchers to produce a rapid/systematic review
d) Collaborating with researchers to developed or implemented a research project
e) Collaborating with researchers to produce a grant application
f) Contributing to the analysis and/or writing up of research publications
g) Attending forums (e.g., conferences, symposia, seminars, meetings) to hear about relevant research findings
h) Engaging in acquisition efforts with researchers to obtain relevant research (e.g., through advisory panels, meetings, committees, focus groups, direct and ongoing contact)
i) Inviting researchers to provide expertise or advice in a personal capacity (e.g., as a policy advisor, as part of an advisory group; steering committee, or secondment)
